# Different cadaver astigmatan mites (Arthropoda: Acari) are designed to bite flesh differently

**DOI:** 10.1007/s00114-026-02108-0

**Published:** 2026-04-29

**Authors:** Clive E Bowman, M Alejandra Perotti

**Affiliations:** 1 Mathematical Institute, University of Oxford, Oxford, OX2 6GG United Kingdom; 2 Ecology and Evolutionary Biology Research, University of Reading, Reading, RG6 6AH United Kingdom

**Keywords:** Acariformes, Chelicera, Ecomorphology, Forensic acarology, Tools, Wasserstein distance

## Abstract

How dead bodies decay is useful forensically. Necrophagous astigmatan mites (Acari: Sarcoptiformes) commonly attacking cadavers change from protein-seeking Type 1 surface feeding omnivores to interstitial Type 2 fragmentary feeding obligate fungivore / microbiovores as human body decomposition progresses after death. An analytical occlusive method shows that at each forensic decay stage the chelae of the astigmatans are designed to bite foodstuff differently. Fixed chelal digits are more ‘toothy’ than moveable digits in such sarcoptiform mites. Variation in fixed digit design is all about the size and pattern of peaks (‘peakiness’) for biting, while astigmatan moveable digit variation is mainly about the size and pattern of valleys (’gullet-ness’) for the ‘scooping’ of decaying material. Foodstuff caught on the moveable digit is thus masticated against the fixed digit like an ‘excavation-bucket’ machine used for handling aggregates in building construction. *Lardoglyphus zacheri* has a distinct chela suitable to slice flesh and grip myofibrils. *Acarus siro* through *Sancassania berlesei* to *Tyrophagus putrescentiae* show a cline in digit design from a dry material ‘demolition crusher’ with rough surface digits, through a wet chunk and slice feeder, to a specialist multifunctional saprophage. *Sancassania berlesei* is unlikely to actively burrow into flesh. Trophic niche width increases from stage 2 (bloated putrefaction) as soft food specialist species invade. Niche width markedly widens at stage 5 (mummified dry desiccated bones and remains), as incorporation into a soil with diverse saprophagous mites is coming to completion.

## Introduction

Forensic acarology provides valuable methods and insights for legal investigations (Perotti and Braig 2022). Much is already known about cadaver decomposition at a general level (Leclercq 1974; Leclercq and Verstraeten 1988). Indeed, Braig and Perotti (2009) in their key review outline a succession of astigmatan mites on carcasses over time so indicating that they might be useful in body staging (OConnor BM 2009, where effectively mites are used as an archaeological tool Baker 2009). Such astigmatans common in farming and occupational environments (Solarz et al. 2022) are generally considered to be *r*-strategists. One limitation of that key review is that it did not distinguish infestors of surface and shallowly buried bodies versus those found in very deep internments. Irrespective of this subtlety, in principle a carcass starts life as a high protein, high fat depot of potential nutrition for a necrophilous astigmatan. The end goal of a body’s decomposition is a dispersed biome approximating soil conditions dominated by nematodes, fungi, bacteria, protists etc., all together as food sources for other animals. It is assumed that cadaver protein and lipid sources would be exhausted by then.

Different astigmatans occur in cadavers at different stages of decomposition (Braig and Perotti 2009). However, are differently designed astigmatan mites involved? How an animal bites is important in their processing of food material. Much work on vertebrate jaws has already been done (Jónsdóttir et al. 2026). Astigmatans have opposable digits to their chelae which function like jaws in food processing. Johnston (1965) and Akimov (1985) illustrates many of these. Which human scale tools do the designs of their cheliceral chelae approximate (Bowman 2025b)? Could they bite flesh differently?

Recently, Bowman (2024b) presented a distance-based method for comparisons of whole chelal moveable digit profiles amongst free-living saprophagous astigmatan mites found in beehives. This ‘edge superimposition’ method of observed forms registered to a reference basis represents a co-planar set of 2 landmarks and $(p-2)$ ‘semi-landmarks’ as Bookstein co-ordinates (Bookstein 1986). The horizontal alignment and adductive output lever moment arm scaling produces a biomechanically-informed one dimensional *p*-tuple ($y_{i},i=1...p$) or row vector (*y*), not requiring a Procrustean adjustment. Bowman (2025a) gave results comparing a *p* by *p* quadratic form ($\Omega =y^{T}.y$) of this data vector for those astigmatans found in bird nests, all as a means to understand potential trophic competition (or the lack of it) by co-inhabitant mites. Using the trophic morphology data in Bowman (2021a) and new digit profile measurements, this paper dissects what astigmatans might be contributing at each of the five dead body decay stages answering a specific set of biologically relevant questions. The type of biting function will be objectively mapped to the degree of opposability of teeth and gullets within and between different species exploiting different niches using an analytical occlusive method. In doing so, it is important to investigate how taxonomic affinity might relate to trophic distinctions.

**Table:** Glossary of specialist terms and abbreviations

Abbreviation	Meaning
$\alpha$	Subtended angle for chela ex (Akimov and Gaichenko 1976)
$\sigma ^{2}$	$\tfrac{1}{i_{e}}\cdot \sum _{i=1}^{i_{e}}(y_{i}-avg(y))^{2}=R_{q}^{2}-(avg(y))^{2}$ (where $avg(y)=\tfrac{1}{i_{e}\cdot \sum _{i=1}^{i_{e}}y_{i}}$, Bowman (2024a))
ATB	Alternate top-bevel like teeth, Bowman (2026)
*CHI*	Cheliceral height index (Bowman 2021a)
*CLI*	Reach or cheliceral length index (Bowman 2021a)
durophagy	The eating behaviour of animals that consume hard-shelled or exoskeleton-bearing organisms
*F*2*AV*	Occluding chelal crunch force (*VR*.*F*1*AV* in Bowman (2020))
FT	Flat top teeth, Bowman (2026)
*IL*	Idiosomal length index (Bowman 2021a)
Kerf	A ‘slit’ in material made for instance by cutting with a saw (Bowman 2026)
*L*1*U*	Adductive input moment lever arm (Bowman 2021a)
*L*2*F*	Reference axis of fixed digit tip to condyle
*L*2*M*	Adductive output moment lever arm (moveable digit tip to condyle) (Bowman 2020)
*MvG*	Food fragment volume of a chelal bite or ‘grab’ at its maximum effective gape (Bowman 2023)
$\Omega$	Summary avSSCP matrix of profile heights
overbite	Fixed digit tip overhangs more anteriorly than moveable digit tip (Bowman 2025b)
psd	Positive semi-definite matrix (https://en.wikipedia.org/wiki/Definite_matrix)
$R_{q}$	Tribological *Root Mean Square* (Bowman 2024a)
RH	Relative humidity
$Root\ Mean\ Square$	$\sqrt{\tfrac{i}{i_{e}}\cdot \sum _{i=1}^{i_{e}}(y_{i})^2}$
*tel*	Stretch, as a profile point process, estimated by $\sum ^{i_{e}}_{i=1}|y_{i}|$ (Bowman 2024a)
*TMvG*	Food fragment size after hypostomal truncation (= pre-ingestion morsel size) (Bowman 2023)
underbite	Moveable digit tip protrudes anterior of fixed digit tip (Bowman 2025b)
*VR*	Adductive moveable digit lever arm velocity ratio $\frac{L1U}{L2M}$ or mechanical advantage (Bowman 2020)
W	Digit depth at the posterior end of the tooth row ($x_{i_{e}}$, Bowman (2024a))
$x_{i_{e}}$	End of mastication surface ($i_{e}$) distance along *L*2*M* reference axis towards condyle (Bowman 2023)
*xRp*	Position of max peak along reference axis, Bowman (2024a)
*xRv*	Position of max peak along reference axis, Bowman (2024a)
$y_{i}$	Profile height above reference axis $i=1...18$ (Bowman 2023)

## Materials and methods

Abbreviations and specialist terms are listed in the Glossary.

**Fig. 1:**
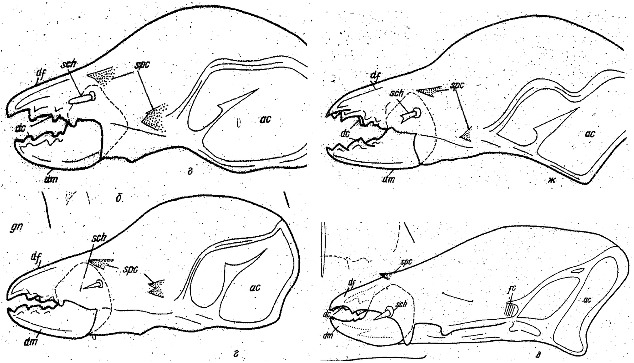
Typical astigmatan cheliceral chelae reproduced from Akimov (1985) with permission showing a variety of digit forms with both teeth and gullets. Top row. Left *Acarus siro* (note fixed digit ‘overbite’). Right *Tyrophagus putrescentiae*. Bottom row. Left *Sancassania berlesei*. Right *Lardoglyphus konoi*. ac = basal articular opening of chelicera. dc = chelal digit teeth. df = fixed digit. dm = moveable digit. sch = cheliceral seta. spc = cuticular spine

Non-histiostomid mite species per cadaver decomposition stage were extracted from Table 3 in Braig and Perotti (2009). Persistent species were any that occurred at two or more decomposition stages. Trophic parameters were taken from Bowman (2021a). Mites listed as genera only in Braig and Perotti (2009) were mapped to all example species belonging to that genera in Bowman (2021a). *Acarus siro* listed in Braig and Perotti (2009), Perotti et al. (2010) was taken to match the trophic design of *Acarus siro* A10B in Bowman (2021a). *Lardoglyphus zacheri* listed in Braig and Perotti (2009), Perotti et al. (2010), Bordas et al. (2020) was taken to match the trophic design of *Lardoglyphus zacheri* L3 in Bowman (2021a). *Sancassania berlesei* listed in Braig and Perotti (2009), Perotti et al. (2010) was taken to match the trophic design of *Sancassania berlesei* C3 in Bowman (2021a). *Tyrophagus putrescentiae* listed in Braig and Perotti (2009), Perotti et al. (2010) was taken to match the trophic design of *Tyrophagus putrescentiae* [A] T13 in Bowman (2021a). Akimov (1985) illustrates the chelicera of three of these species and the closely related *Lardoglyphus konoi* (Fig 1).

**Table 1:** Free-living astigmatans used grouped by family and genus (collection details in Bowman (2021a)) commonly found in cadavers. *Tyrophagus* breeding group [...] follows Griffiths (1979). ’Presence in other habitats’ records from Hughes (1976) not including feathers, nor fruit. Number string: 1 = positive, 0 = negative, in the order: Bat roosts, Mammal nests, Mattresses, Dust, Broiler houses, **Cadavers** (Braig and Perotti 2009), Meat, Cheese, Storage, Grassland. Population = code ex Bowman (2021a). $\dag$ = Omnivore (others are Fragmentary feeders). * = Surface habit (others are Interstitial)

Family	Taxon	Presence	Population	Origin
		in other	code	
		habitats’		
Acaridae				
	Acarinae			
	*Acarus siro*	00011**1**0110	A10B *$\dag$	Pigmeal. GB. 1940‘s
	*Tyrophagus putrescentiae* [’A’]	00000**1**0110	T13	Groundnuts. South Africa. 23//7/68
	Rhizoglyphinae			
	*Sancassania berlesei*	00001**1**0110	C3 *$\dag$	Fishmeal. Karachi, Pakistan
Lardoglyphidae				
	*Lardoglyphus zacheri*	00000**1**1000	L3 *	Mexican Daphnia Meal. Manchester, GB. 5/10/79

Out-group comparison species included *Acarus siro* A15, *Tyrophagus brevicrinatus* T89, *Tyrophagus neiswanderi* T6, *Tyrophagus perniciosus* T8 & T38, *Tyrophagus tropicus* T90, *Dermatophagoides farinae* D4, and  *Dermatophagoides microceras* D5 from Bowman (2021a).

Twenty female adults of each species of free-living saprophagous astigmatan mites common in cadavers were examined (originating from the wild and kept as live cultures at the now defunct Pest Infestation Control Laboratory, Slough, UK, Table 1). These had been collected, identified and nurtured by the late Dr Donald Griffiths and his team investigating principally their taxonomy. They are referred to by their original ‘short-hand’ code so as to allow traceability back to source and comparison to existing publications featuring them. The *Tyrophagus* spp. breeding groups follow Griffiths (1979). Alcohol preserved samples of the mite cultures have been deposited at: the British Museum (Natural History), London UK under accession number AQ ZOO 2020-78, and the Museum of Zoology, University of Michigan, Ann Arbor, MI USA under accession number ITR-UMMZ-I-2020-018.

Mites were cleared in lactic acid, mounted on microscope slides and examined by Nomarski interference phase-contrast light microscopy. Calibrated drawings were made and data extracted from them using ImageJ 1.53k (http://imagej.nih.gov/ij).

The mastication surface of the grasping chela of each mite was estimated as the length of the moveable digit output moment lever arm (*L*2*M* in $\mu$m) minus the length of the adductive input moment lever arm (*L*1*U* in $\mu$m), Bowman (2023). Moveable and fixed digit profile heights ($y_{j,k}$ where $j=1...20, k=2...18$) for each digit were characterised from preserved samples using the methods of Bowman (2023, 2024a, 2024b, 2025a, 2026). Note that the maximum effective gape angle $\delta =cos^{-1}\frac{(L2M-x_{i_{e}})}{L2M}$ and not as printed in Bowman (2023).

The method to estimate $\Omega$ matrices is comprehensively explained in Bowman (2024b, 2025a). Briefly, define a 17 x 1 column vector $$y_{i,j}=\left( \begin{array}{c} y_{i,j,2} \\ \vdots \\ y_{i,j,18} \\ \end{array} \right)$$ for the measurements of the *j*th individual of the *i*th taxon, where the third index is the profile location number. Assemble these columns over the $n_{i}$ individuals for that taxon by defining

$$Y_{i}=\left( \begin{array}{cccc} y_{i,1,2}& y_{i,2,2}& ...& y_{i,n_{i},2} \\ \vdots & \vdots & ...& \vdots \\ y_{i,1,18}& y_{i,2,18}& ...& y_{i,n_{i},18} \\ \end{array} \right)$$

and calculate the symmetric matrix $\Omega _{i}=\frac{1}{n_{i}}Y_{i}.Y_{i}^{T}$ (where $^{T}$ means transpose), which herein is a 17 x 17 average non-scaled non-centred ‘sums of squares and cross-products’ (SSCP) matrix for the *i*th taxon as the tip ($p=1$) is omitted since it is zero height by definition. It can be thought of as a matrix analogue of a positive real number. As such it can be a convenient data summary to make overall comparisons for morphologists. Note that this is not a scaled CSSCP of normalised variates i.e., neither mean correction nor variance scaling was done. A pre-multiplier of $\frac{1}{n}$ was used for each matrix where $n=$ number of mites used for that matrix ($n=20$ in this paper). The SSCP is explicitly labeled as ‘average’ here because *n* could in theory vary from collection to collection. This approach retains the mean profile size for each taxon and allows larger variance measures to be deemed as more important in any comparisons. No Procrustes transformations were employed to yield these average SSCP $\Omega _{i,k}$ matrices (where $i=$taxon and $k=$ replicate of that *i*th taxon).

All data manipulations were carried out in Excel for Mac version 16.78.3 and R version 4.3.1 (2023-0616) ‘Beagle Scouts’. Unweighted Manhattan distance was used to compare the trophic design differences between the key species using Table 11 ’High-throughput screening field-use look-up’ in Bowman (2021a).

The Burres-Wassersetin distance between two full rank positive semi-definite matrices (psd) *A* and *B* is given by the closed form (Bhatia et al. 2017 eqn 1)

$$\delta (A,B)=[tr(A)+tr(B)-2tr((A^{1/2}BA^{1/2})^{1/2})]^{1/2}$$

where *tr*() means ‘trace’ of the matrix, or

$$\delta ^{2}(A,B)=tr(A)+tr(B)-2tr(\sqrt{[\sqrt{A}B\sqrt{A}]})$$

Mohan (2023). It is a Riemannian manifold metric (Bhatia et al. 2017). For a square matrix the trace is a scalar equal to the sum of the elements on its main diagonal, from the top-left to the bottom-right. The trace can also be found by summing the matrix‘s eigenvalues (counted with multiplicities).

A geodesic curve ($0\le t \le 1$) *on this metric* between matrices *A* and *B* is given by

$$\gamma (t)=(1-t)^{2}A+t^{2}B+t(1-t)[(AB)^{1/2}+(BA)^{1/2}]$$

and is denoted $A\diamond _{t}B$ Bhatia et al. (2017) eqn 39. This is bounded for $0\le t \le 1$ as

$$A\diamond _{t}B \le (1-t)A + tB$$

Bhatia et al. (2017) eqn 52. The midpoint (i.e. $t=\frac{1}{2}$) is the Wasserstein mean of matrices *A* and *B* =

$$\frac{1}{4}[A+B+(AB)^{1/2}+(BA)^{1/2}]$$

Bhatia et al. (2017) eqn 48. Further details can be found in Bhatia et al. (2019).

**Fig. 2:**
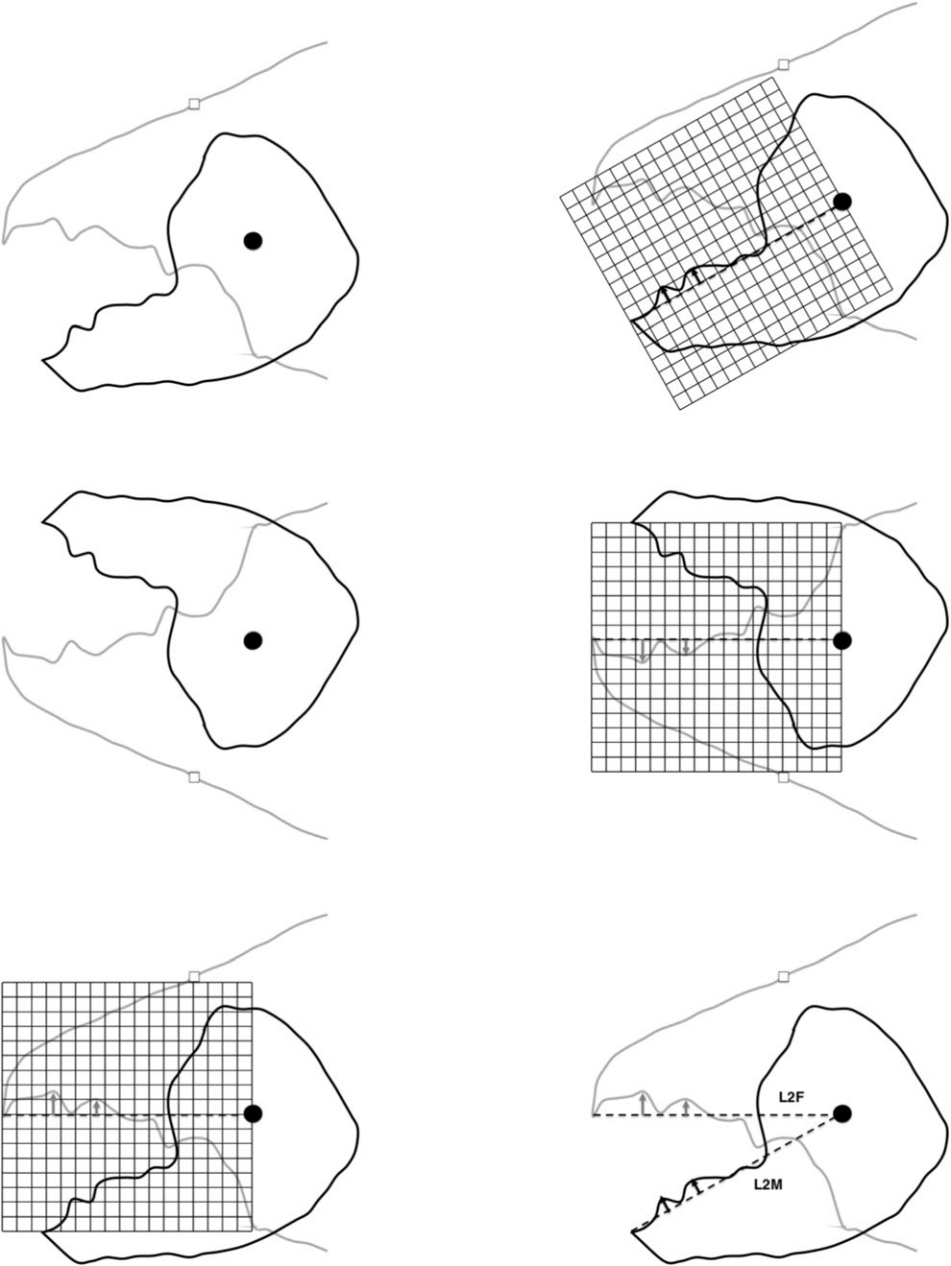
Scoring of profile peaks (i.e., location vector $x_{j}$ and height vector $y_{j}$) for each digit using open chela of *Tyrolichus casei* for ease of illustration. Top row. Left, open chela, moveable digit in black, fixed digit in grey. Articulating condyle as black dot. Right, reference axis *L*2*M* from moveable digit tip to condyle as dashed line plus equispaced grid for profile sampling. Note black arrows indicating location ($x_{i}$) and height ($y_{i}$) for the first two moveable digit peaks posterior of moveable digit tip. $x_{i_{e}}$ is near sample location $i=12$. Middle row. Left, chela flipped vertically. Right, reference axis *L*2*F* from fixed digit tip to condyle as dashed line, equispaced grid and grey arrows indicating location ($x_{i}$) and height ($y_{i}$) for the first two fixed digit ‘gullets’ posterior of fixed digit tip. $x_{i_{e}}$ near sample location $i=13$. Bottom row. Left, re-flipped chela, mapping fixed digit gullets to fixed digit ‘peaks’. Right, final montage showing how the profiles can approximately fit together on moveable digit occlusion (peak to gullet, gullet to peak). Similar approach is done to score locations of true gullets $x_{j}$ and for their heights $y_{j}$ and at the 18 equispaced reference axis increments. Note $L2M\ne L2F$. All measures in $\mu$m

The one dimensional ‘earth-mover’ variant of this was estimated using *wasserstein1d* in the R library *transport* (with $p=2$). The location ($[x_{i,j}]$, $0<x_{i}\le x_{i_{e}}$) along the reference axis of digit tip to condyle and the perpendicular height of all mastication surface peaks and gullets ($y_{i,j}$) to their reference axis scored for each digit (e.g., Fig. 2) within the *j*th individual in $\mu$m were sorted together by $x_{i}$ value ($1<i<18$ for each individual) separately for each digit. The two resultant $x_{j}$ vectors were compared to each other with their corresponding $y_{j}$ vector for the moveable digit and their $-y_{j}$ vector for the fixed digit, as weights within each individual. Note that fixed digits weights were finally adjusted before use by subtracting $min_{i}(y_{i,j})$ (to ensure $\ge 0$) and normalisation occurs within the R routine.

The resultant calculated 2-wasserstein distance represents the work needed to ‘move’ the empirical distribution of peaks from the configuration on one digit optimally to that on the other digit for the *j*th individual. A second run of vertically reflected data (i.e., $y_{i,j}\rightarrow [ \max _{i}(y_{i,j})-y_{i,j}]$) was made to calculate the work needed to ’move’ the empirical distribution of gullets from the configuration on one digit optimally to that on the other digit for the *j*th individual. These latter two distances are different, one focusing on peak differences (’peakiness’), the other on gullet differences (’gullet-ness’). They were averaged to produce an overall summary measure per individual for all asperities. Posed as in the preceding paragraph, low distances are a ‘good’ reciprocal fit of asperities between the two digits, high distances indicate specific differentiation between the two digits. This transporting formulation thus allows for location changes and normalised specific height changes within location for each individual mite (much as evolution would be expected to act on the *pattern* of a continuous morphological profile). The only assumptions are that the inherent ‘deformation’ process is genetically based, is smooth and common (but of different magnitude) between individuals and there is a selective advantage for the occlusive pattern. The fit or lack of fit may be superseded by the advantage of a single digit’s function (c.f., underbite - see Bowman (2025b)).

Multidimensional scaling ordination was done using *mds* in library *smacof* in R. As this 2-wasserstein distance is not a similarity measure, being a metric distance itself and over modest scale changes in this study, there was no need to transform this by a suitably scaled $\sqrt{(1-s)}$ adjustment (Nader et al. 2019), although that would make it more Euclidean in nature.

## Results and Discussion

### Which are the key species in cadavers and what is their relationship?

**Fig. 3:**
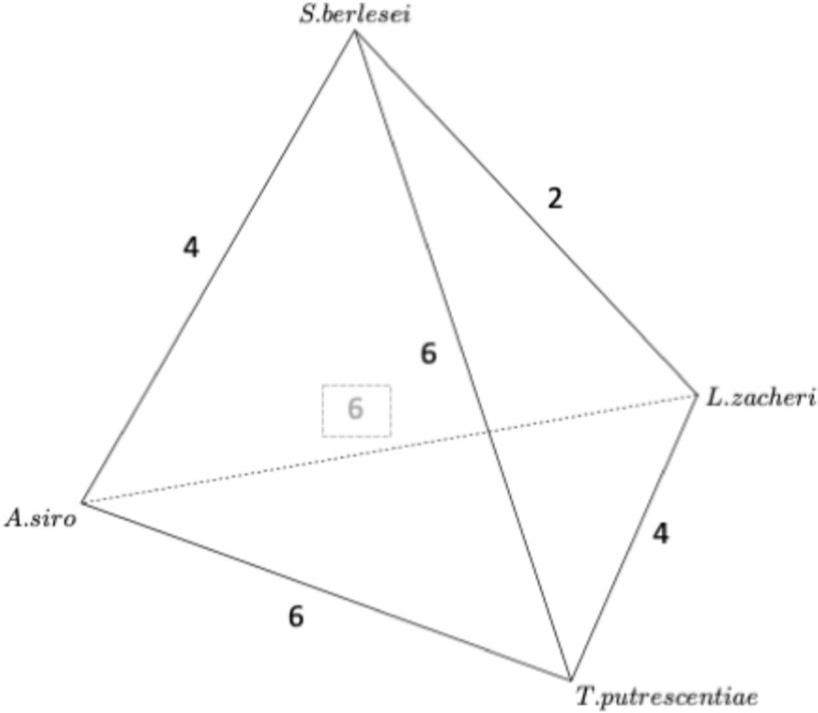
Network based upon number of trophic character changes (from Bowman 2021a) between astigmatan species that are persistent during carcass decomposition. Note the graph is not planar

From Braig and Perotti (2009), Perotti et al. (2010), four astigmatan species persist across multiple stages of decomposition: *Acarus siro*, *Lardoglyphus zacheri*, *Sancassania berlesei*, and *Tyrophagus putrescentiae*. In terms of the changes one to another in trophic characters Fig. 3 shows their interrelationships. *Acarus siro* and *Tyrophagus putrescentiae* occupy distinct niches being the furthest away from the others (and each other) on average. The other two species share somewhat stronger commonalities.

**Fig. 4:**
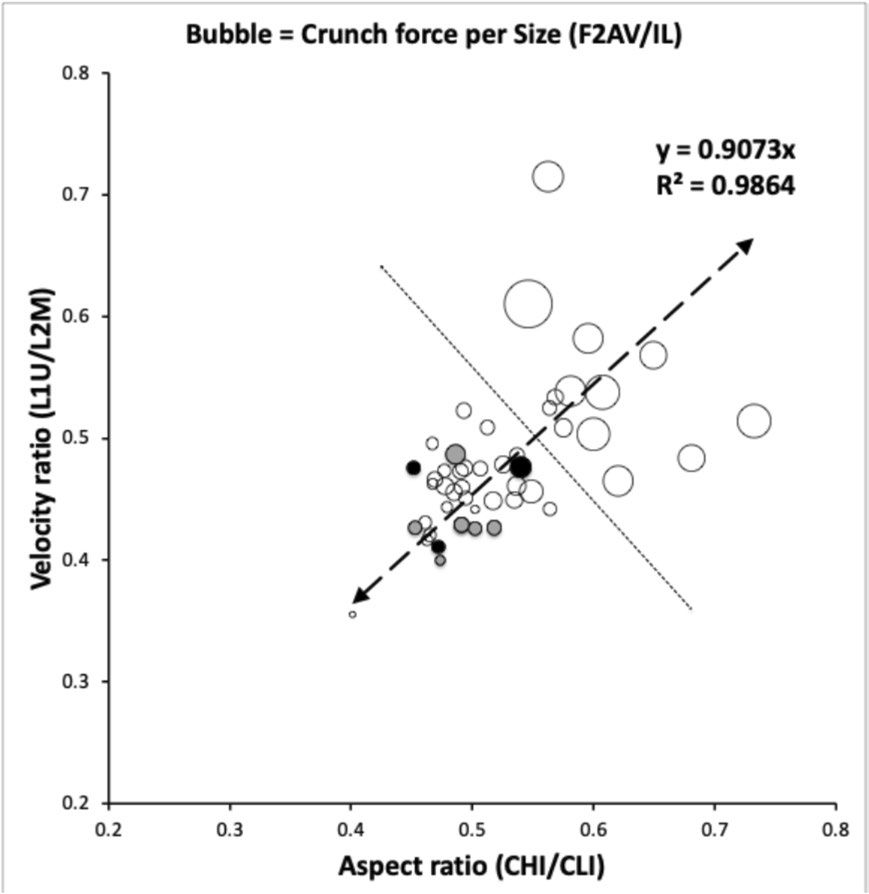
Astigmatan cheliceral and chelal design space amended from Bowman (2021a) under Creative Commons permission. Black dots = cadaver-inhabiting species. Grey dots = species of same genera as the cadaver species as comparators. Open dots = remaining free-living non-cadaver-inhabiting astigmatans

All four (and out-group comparators) belong to the lower groupings in Fig. 4 (and Fig 25(c) of Bowman 2021a) i.e., they are not adapted for consuming very hard foods. All four are approximate overall ‘shrunken/swollen’ versions of each other (Bowman 2021a). *Tyrophagus putrescentiae* is microsaprophagous, the remaining three species macrosaprophagous. *Sancassania berlesei* like *Acarus siro* is a pan-saprophage. Note that the surface feeding *Tyrophagus longior* is also known from cadavers (Perotti 2009).

The presence of *Sancassania* species in the late stages of decomposition (mainly mummified corpses) has been described since the early works of Pierre Mègnin (Mègnin 1894; Perotti 2009) and Marcel Leclercq (Leclercq 1974; Leclercq and Verstraeten 1988). The association of *Sancassania* species with advanced decay and mummified remains seems to prevail over time, with recent case work confirming the dominance of *Sancassania* as the main (mite) scavenger (Saloña et al. 2010; Szelecz et al. 2018). In addition to much unpublished experimental and case work Perotti and co-workers often found *Sancassania berlesei* or *Sancassania mycophagus* in hidden, concealed corpses or enclosed burials with locally very high humidity conditions ($RH = 95-100\%$) - a particular requirement of the genus. Accordingly such species should be pulled out from Fig. 3 which then makes the graph of trophic inter-relationships planar (yet still retains the *Acarus siro* distinction). Note that covered, wrapped conditions also foster the occurrence of filter feeding anoetids on bodies.

### Do mites switch to burrowing in the carcasses as decay increases?

**Fig. 5:**
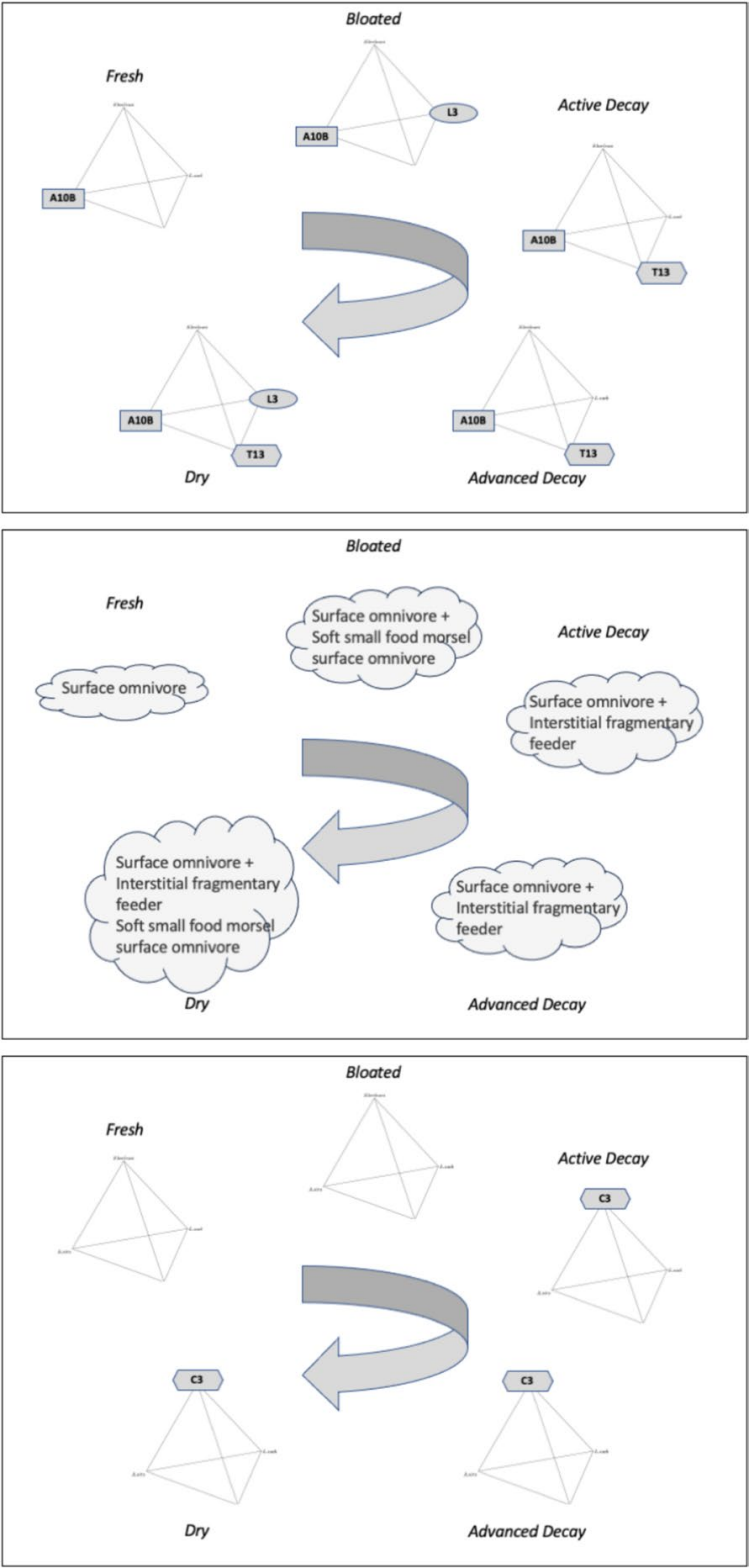
Time course of astigmatan mites infesting cadavers. A10B = *Acarus siro*. C3 = *Sancassania berlesei*. L3 = *Lardoglyphus zacheri*. T13 = *Tyrophagus putrescentiae*. Upper: Incidence of species that are persistent during surface or shallow buried carcass decomposition (clockwise transit over time) from Braig and Perotti (2009). Middle: Trophic design class thereof. Surface omnivores are of ’Type 1’ trophic design, Interstitial fragmentary feeders are of ’Type 2’ trophic design Bowman (2021a). Height of cloud represents apparent niche width. Bottom: Incidence for astigmatan species that are persistent during high humidity non-ventilated carcass decomposition (clockwise transit over time). *Sancassania berlesei* has a distinctly different surface omnivore feeding habit compared to *Acarus siro* (Bowman 2021a)

Only in surface or shallow burials does the incidence of the four persistent species change over time (Fig. 5).

Surface feeding omnivores are found in the initial fresh all the way to the dry stage of decay. The first specialist (*Lardoglyphus zacheri*) as a soft small food morsel surface feeder (Bowman 2021a) appears at the end of the putrefying bloated (gas swollen, odour of decaying flesh) stage to accompany the demolition/excavating chunk feeding species *Acarus siro*. Interstitially feeding species (i.e., *Tyrophagus putrescentiae*) appear first at the active decomposition stage and remain to the end of carcass processing (Perotti et al. 2010).

Goff (2009) shows that the active decay stage begins when the skin is broken (offering much easier trophic access). Carcass flesh is of a creamy consistency at this point with exposed parts black and putrefying. The body collapses as gases escape giving a very strong odour of decay around this colonisation with mites of a different trophic design. As befits its habit in high humidity carcasses from the active decay stage forward (Fig. 5), *Sancassania berlesei* has a distinct feeding habit compared to the demolition feeder *Acarus siro*. It is known to be a large nematode feeding necrophagous mite (Bowman 2021a). Chmielewski (2003) contains a comprehensive list of its biome and feeding preferences.

Regarding burrowing, Bowman (2023) estimates that for the interstitially feeding *Tyrophagus putrescentiae* in UK bee hives, with a mean bite volume ($Mv_{G}=4703\mu$m$^3$, $TMv_{G}=4246\mu$m$^3$) this might allow it to excavate its own body size within foodstuffs in about 94 mins on average. In this study herein Table 2 shows similar values for *Tyrophagus putrescentiae* T13.

**Table 2:** Mean bite volumes estimated for astigmatans commonly found in cadavers (see Bowman 2023). Estimated excavation time based upon average idiosomal index values from Bowman (2021a)

Taxon	MvG	TMvG	Excavation time
	($\mu$m$^3$)	($\mu$m$^3$)	(mins)
*Acarus siro* A10B	7180	6349	40
*Tyrophagus putrescentiae* T13	4773	4435	97
*Sancassania berlesei* C3	3022	2670	119
*Lardoglyphus zacheri* L3	3646	3211	48

Compared to the surface omnivore *Acarus siro*, known to excavate wheatgerm from grains, the fragmentary feeder *Tyrophagus putrescientae* may not be a strong burrower despite its interstitial habit. *Tyrophagus putrescentiae* (and eventually in carcasses *Tyrophagus longior*, Perotti 2009) may thus rely upon others to open up the dead body by trituration before later stage colonisation by it (i.e., active stage $\rightarrow$ dry, Fig. 5). The surface habit *Lardoglyphus zacheri* L3 is confirmed as a likely burrower commensurate with its early appearance at the ‘bloated’ stage. *Sancassania berlesei* C3 appears to be a surface habit ‘grazer’. More detailed investigations *in situ* as to their activity at a micro-scale are needed.

### Does the ability to process protein change?

*Acarus siro* is known to have high levels of protease, *Tyrophagus putrescentiae* low values Bowman (1981), although Erban et al. (2015) found that high-fat, high-protein food accelerated *Tyrophagus putrescentiae* population growth compared with the high-carbohydrate diet. While comparable enzyme figures are not available for *Lardoglyphus zacheri* or *Sancassania berlesei*, their position on the food toughness display of Fig 29 in Bowman (2021a) strongly suggests that at least *Sancassania berlesei* should have an elevated protease activity, confirming the observed fall over time between (only) *Acarus siro* at the fresh initial decay stage to the hypocarnivorous *Tyrophagus putrescentiae* at the remaining active $\rightarrow$ advanced $\rightarrow$ dry decay stages, Fig. 5. This is consilient with the progressive exhaustion of cadaver proteins over time.

### Is the switch of feeding over time confirmed by other mites that have been found?

**Fig. 6:**
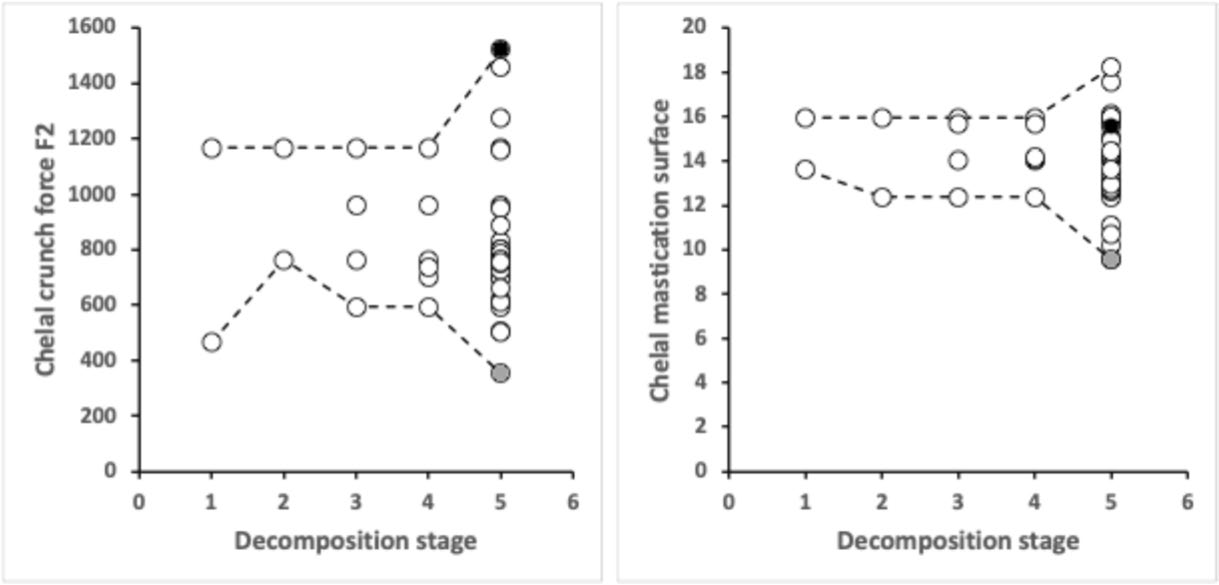
Change in chelal adductive ’crunch force’ *F*2 (Left) and approximate chelal mastication surface size (Right) of all astigmatan species (open circles) found in carcasses by decomposition stage 1-5 (fresh, bloated, active, advanced, dry) listed in Braig and Perotti (2009). Black circle = *Lepidoglyphus destructor*. Grey circle = Winterschmidtiidae. Maximum (upper) and minimum (lower) dashed lines for illustration

Figure 6 shows what happens for two key trophic parameters (chelal ’crunch force’ and approximate mastication surface size) when all records of mite species in Braig and Perotti (2009) are included. Omitting *Sancassania berlesei* has no effect upon the enclosing ‘maximum-to-minimum’ envelopes for the data over the decay stages.

From when the carcass macroscopically changes (i.e., bloating stage 2, Goff (2009)), mites with a lower and lower chelal bite force deliverable by their chelicerae are found (cf. the minimum decreases with decay stage on left of Fig. 6). This is consilient with body tissue breakdown/catabolism. At the mummification stage, acarine specialists packing an even more powerful punch needed for biting very hard desiccated food are present (e.g., *Lepidoglyphus destructor* G6 Bowman 2021a). So, niche width is markedly wider than before on both counts. Chelal velocity ratio in itself was not informative. Note that although *Sancassania* is known from mummies (Mègnin 1894), the chelal crunch force for *Sancassania berlesei* determined by Bowman (2021a) was not above average for its body size (*plot not shown*).

Similarly the ability to handle different varieties of sizes of food suddenly explodes at dry decay stage 5 (indicative of a diversified mature ’climax’ food source like soil beginning to appear). The two species with the highest chelal mastication surface values at stage 5 in Fig. 6 right are *Rhizoglyphus* spp., these are soil mites that feed on decayed roots and bulbs (Rai et al. 2020).

The trophic categories of the extra species in Fig. 6 cover both extra macro-saprophagous and micro-saprophagous forms and were

- *Stage 1, fresh decay*: + 1 x Interstitial Fragmentary feeder (Type2 = obligate fungivore / microbiovore) i.e., *Acarus farris*.
- *Stage 4, advanced decay*: + 1 x Surface fragmentary feeder + 1 x Interstitial Fragmentary feeder (Type2 = obligate fungivore / microbiovore).
- *Stage 5, dry decay*: + 4 x Surface Omnivore (Type1) forms + 1 x Small food morsel surface omnivore + 1 Soft small food morsel surface omnivore i.e., *Tyrophagus longior* + 1 Soft food (large nematode) surface omnivore + 4 Surface fragmentary feeder forms (including *Acarus immobilis*) + 1 Soft food (large nematode) interstitial omnivore + 1 x Interstitial omnivore + 11 x Interstitial Fragmentary feeders (Type2 = obligate fungivore / microbiovore).

This increased diversity of habit confirming the widening of the trophic niche.

One of these extra species is *Tyrophagus longior*. It is usually considered as a pest of plants. Its data sits approximately at the average values for dry decay stage 5 in both panels of Fig 6. As there are no plant tissues in a carcass, what could *T.longior* be doing? Could it be consuming nematodes like some other free-living astigmatans do? Soil nematode community analysis has been used to help infer the Post-Mortem Interval in human bodies Szelecz et al. (2018), but those authors do not mention *Tyrophagus longior* being present alongside them. Szelecz et al. (2018) lists the common soil bacterial feeding Cephalobidae, Rhabditidae and Plectidae as the three most abundant nematode families associated with a long-term carcass (as well as some different families found away from the actual cadaver). Might *Tyrophagus longior* specifically feed on any of these? Laboratory feeding tests are needed. Future detailed field work could also confirm whether the transit from advanced decay stage 4 to dry decay stage 5 is the time of major invasion (into carcasses) of the specific nematode species likely to be consumed by the herbivorous pest *T. longior*. Otherwise this acarid (known to be of surface omnivore design for eating little chunks of small food morsels in tiny mouthfuls, Bowman (2021a)) is consuming something else at this late point.

Is this niche widening over time confirmed by other publications? Kaur et al. (2019) examining cattle carcasses lists many of the mites found in Braig and Perotti (2009). Rai et al. (2021) points to pyroglyphids appearing at the active decay and at the dry remains stages in pig decomposition. A durophage is an animal specialised for feeding on such hard material. Some pyroglyphids are even stronger durophages that glycyphagids (Bowman 2021a) so their late-on occurrence makes sense (they are also interstitial in habit). It is not clear if *Glycyphagus domesticus* should be included as a carcass infesting mite or not, terminally a carcass being so dry as to only allow durophagous invaders to prosper.

### Are the trophic changes consilient with physical conditions?

Decomposing bodies produce heat (Leclercq 1974) so it is assumed that temperature conditions remain elevated to encourage some establishment and growth of astigmatan mite populations throughout all the decay stages bar the final denouement (unless strongly cold autumnal or winter conditions arise for fully exposed carcasses). However, as Bowman (2021b) shows for stored product *Tyrophagus putrescentiae* samples, heavy mite infestations probably produce enough heat in themselves to confound strong inference about the externally driven physical temperature conditions where they are.

Assuming that the decaying carcass first increases in liquidity then declines to a desiccated state, does the above successional pattern match known humidity tolerances amongst the mites? *Acarus siro* development was completed only at relative humidities (RH) above 65% (Cunnington 1966) with an optimal humidity of 85% (like that for *L.destructor* too Hubert et al. 2013). Jayas et al. (1992) illustrates 80-90% RH as the optimum. Relative humidities of 65% or greater favour the growth of *T. putrescentiae* (Krishnan et al. 2019) with an optimal humidity of 85% (Hubert et al. 2013). Jayas et al. (1992) illustrates 85-95% RH as the optimum. When feeding on moulds Rivard (1961) found 90% RH to be best for the tyrophagid. This does not suggest that relative humidity is the differential factor between the incidence of these two mites. However, the relative humidity of carcass locations where the mites are actually found needs monitoring so that the pattern could be matched to their preferences over time. Although lardoglyphids are known from desiccated mummies (Baker 2009) and studies of the ecology of *Lardoglyphus zacheri* have been made (Iverson et al. 1996), no comprehensive life-table investigation appears to have been carried out since the early observations of Hughes (1976) at $87\%$ RH.

*Sancassania berlesei*, historically reared on dried yeast, thrives in exceptionally damp food stores where it can be found feeding in liquid films. Sinha (1964) found it to be mycophagous. Whilst its temperature requirements are known (Doaa et al. 2014) when feeding upon nematodes, its relative humidity requirements are not so clear (although Szelecz et al. 2018 states that "...exposure to low humidity levels leads either to death or to moulting into more resistant immature stages commonly known as hypopi". It was reared at $27^{\circ } C$ and $80\%$ RH by Rodriguez and Stepien (1973) who experimentally characterised it at $25^{\circ } C$ and $92\%$ RH. Chmielewski (2003) kept it at the even higher $100\%$ RH. Jayas et al. (1992) illustrates 90-100% as the optimum.

*Sancassania berlesei* is a conundrum. It is found in (deep-litter) broiler houses (Table 1) where dead birds can occur, but this species is not a recognised bird nidicole. Indeed, bird nest humidity is a critical factor in ectoparasite regulation (García-del Río et al. 2024) and egg viability - with nest walls being very leaky to water vapour (Deeming et al. 2020). What is needed is for ecologists to measure the relative humidity in carcasses where adult cadaver mites actually are. Indeed, filter feeding histiostomids would be expected to be found in cadaver fluids during decay stages 2 (bloated), 3 (active) and 4 (advanced). Szelecz et al. (2018) describes the biology of association of *Sancassania berlesei* with corpses in detail. It is thought to be mycophagous but what particular fungi tolerate very high fluid conditions? Are such fungal species preferentially attractive to this mite? *Sancassania* spp. are also known to predate upon nematodes (Karagoz et al. 2007) and forensic investigators do find such nematodes infesting high humidity enclosed cadavers (Szelecz et al. 2018). Exactly how fungi, nematodes and *Sancassania berlesei* interact needs further experimental validation.

### Underbite, overbite, digit tips and digit depth

There was only an exact match of chelal moveable and fixed digit tips observed in 2 out of the 80 specimens examined. The $probability(overbite)=0.75,0.8,0.5, 0.5$ for *Acarus siro* A10B, *Sancassania berlesei* C3, *Lardoglyphus zacheri* L3 and *Tyrophagus putrescentiae* T13 respectively. A value of 0.5 would indicate random chance over measurement error. The average scale of ‘overbite’ was $0.49\mu$m and $0.44\mu$m for *Acarus siro* A10B and *Sancassania berlesei* C3 respectively. That is $\approx 2.6-2.9\%$ respectively of their moveable digit mastication surface $x_{i_{e}}$.

**Fig. 7:**
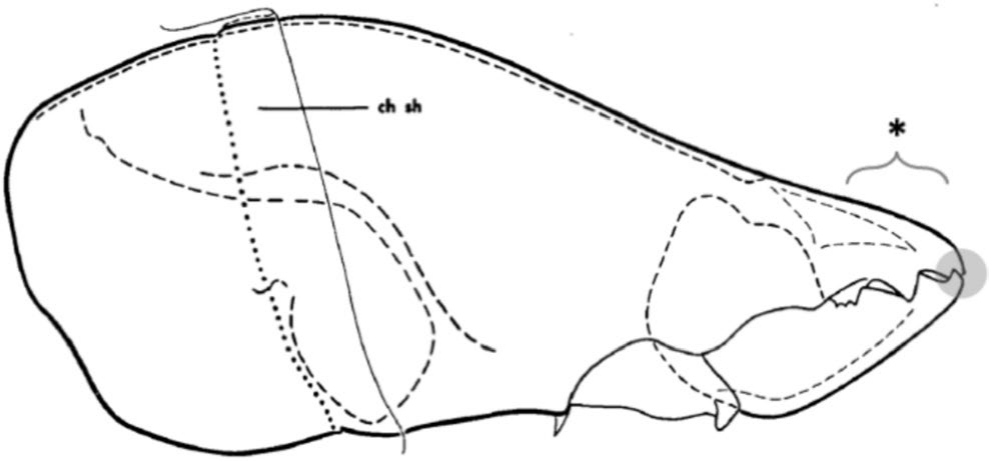
*Sancassania berlesei* chelicera amended with permission from Johnston (1965) under ‘Fair use’ (see https://hsl.osu.edu/form/fair-use-checklist) and with Author’s permission. Grey circle highlights small fixed digit ‘socket’ for moveable digit tip to swing into on occlusion. Note good (peak to gullet, gullet to peak) fit between digit asperities (*). ch sh = cheliceral sheath

Many acarid individuals examined showed evidence of a small fixed digit ‘socket’ into which the moveable digit tip could swing on occlusion: $\frac{18}{20}$ specimens for *Acarus siro* A10B, $\frac{12}{20}$ specimens for *Sancassania berlesei* C3 (Fig. 7), and $\frac{16}{20}$ specimens for *Tyrophagus putrescentiae* T13. The situation for *Lardoglyphus zacheri* L3 was ambiguous. This feature would ensure occlusal locking of opposing chela digits together in the first two species, but must have a different function in *Tyrophagus putrescentiae* T13 given it’s no clear overbite.

**Fig. 8:**
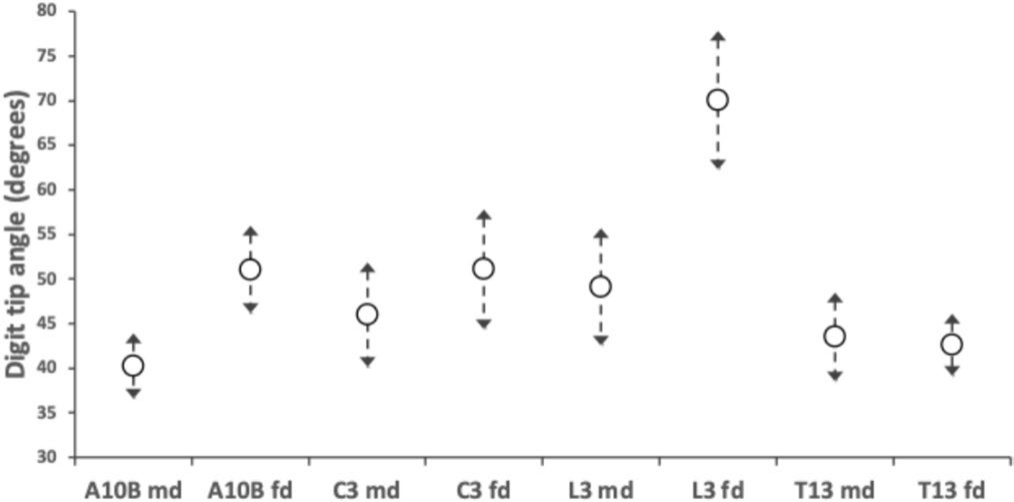
Mean and $95\%$ confidence intervals for digit tip angle (measured in $^{\circ }$ as in Bowman 2026) across astigmatans commonly occurring in cadavers. A10B = *Acarus siro*. C3 = *Sancassania berlesei*. L3 = *Lardoglyphus zacheri*. T13 = *Tyrophagus putrescentiae*. md = moveable digit. fd = fixed digit

Figure 8 illustrates that only *Lardoglyphus zacheri* L3 has any digit tip differentiation from a default scalporial function inferred in astigmatans before (Bowman 2026).

This species may use the tips of its fixed digits to hook into and drag back material having plunged ‘open-mouthed’ down into flesh axe-like. In that way their design approximates ‘seam opening pliers’ (see Fig. 10 in Bowman 2025b).

**Fig. 9:**
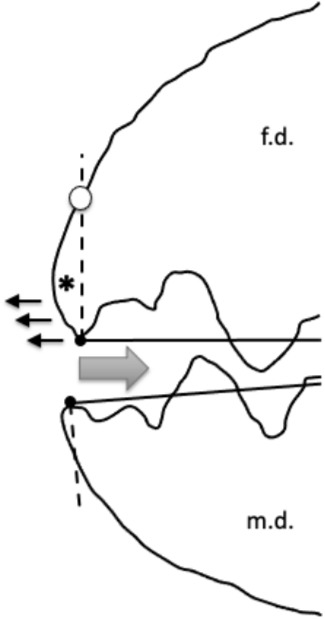
A ‘snub’ nose (*) to a chelal digit resists vertical bending on digit retraction (grey block arrow) in food stuff. Distal part of *Acarus siro* A10B chelicera. md = moveable digit. fd = fixed digit. Black lines joining digit tips to condyle (off page to the right) are *L*2*F* and *L*2*M* with perpendicular dashed lines at digit tips. Open circle is notional point of rotation, here as the fixed digit tip undergoes flexing force (small black arrows)

Having a ‘snub nose’ to a fixed digit may facilitate the drag-back action of chelae (Fig. 9). This also illustrates a possible reason for the excess power law on digit filament strengthening found by Bowman (2024b) for the *Glycyphagus domesticus* moveable digit on sawing retraction. Herein in this study it is shown for *Acarus siro* fixed digit tip scalporially ‘hooking’ material on retraction.

However, subjectively scoring the frequency of fixed digit ‘snub noses’ over the four cadaver species herein does not show any obvious differences (average $\approx 40\%$), although they appear more pronounced in *Acarus siro* A10B and less pronounced in *Tyrophagus putrescentiae* T13. This would suggest that *Lardoglyphus zacheri* chela may in part act like a specialist gleaner (Bowman 2025b), although it lacks clear overbite. Detailed finite element modelling of stresses and strains in chelal digits is needed in follow-up work.

Fixed digit depth (at the end of the mastication surface $x_{i_{e}}$) varies across the four species, *Acarus siro* A10B has the highest ($17.6\mu$m), *Lardoglyphus zacheri* has the lowest ($9.6\mu$m), with the other two species having similar values ($\approx 13\mu$m) in between. At $13.6\mu$m, overall fixed digit depth is approximately $30\%$ higher than that of the moveable digit (at $10.5\mu$m), the fixed digit acting therefore like the strengthened dorsal nail on the fingers of primates to deliver leverage for proprioreception (https://simple.wikipedia.org/wiki/Nail_(anatomy)). Note that if both digits were equally ‘floppy’ there would be poor operating characteristics for touching material.

Fixed digit kerf and fixed digit distal angle are consequently elevated compared to that of the moveable digit by $14\%$ and $16\%$ respectively overall. *Acarus siro* A10B has a noticeably higher kerf value for both digits compared to the other three species ($>30\%$ increase, *plot not shown*). Broad mouths would indicate a major carrying capacity for food in each bite like puffin beaks, or a design for a wide swipe of food material like the gathering of herbage by the lips of a white rhino.

### Do the mites bite differently?

All of the cadaver astigmatans show chelate-dentate chelicerae (Fig. 1). At moveable digit velocity ratio (*VR*) values for *Acarus siro* A10B of 0.476, for *Sancassania berlesei* C3 of 0.429, for *Lardoglyphus zacheri* L3 of 0.476, and for *Tyrophagus putrescentiae* T13 of 0.411, the data is exceedingly similar to that of Bowman (2021a) ($r=0.9999$). Their average $\alpha$ angles of $79^{\circ },82^{\circ },72^{\circ }$, and $83^{\circ }$ respectively for *Acarus siro* A10B, *Sancassania berlesei* C3, *Lardoglyphus zacheri* L3 and *Tyrophagus putrescentiae* T13 confirm for all these cadaver astigmatans that their moveable digits operate like Class 1 lever machines (see Bowman 2025a). The trophic categorisations of Bowman (2021a) are assumed to still apply.

**Fig. 10:**
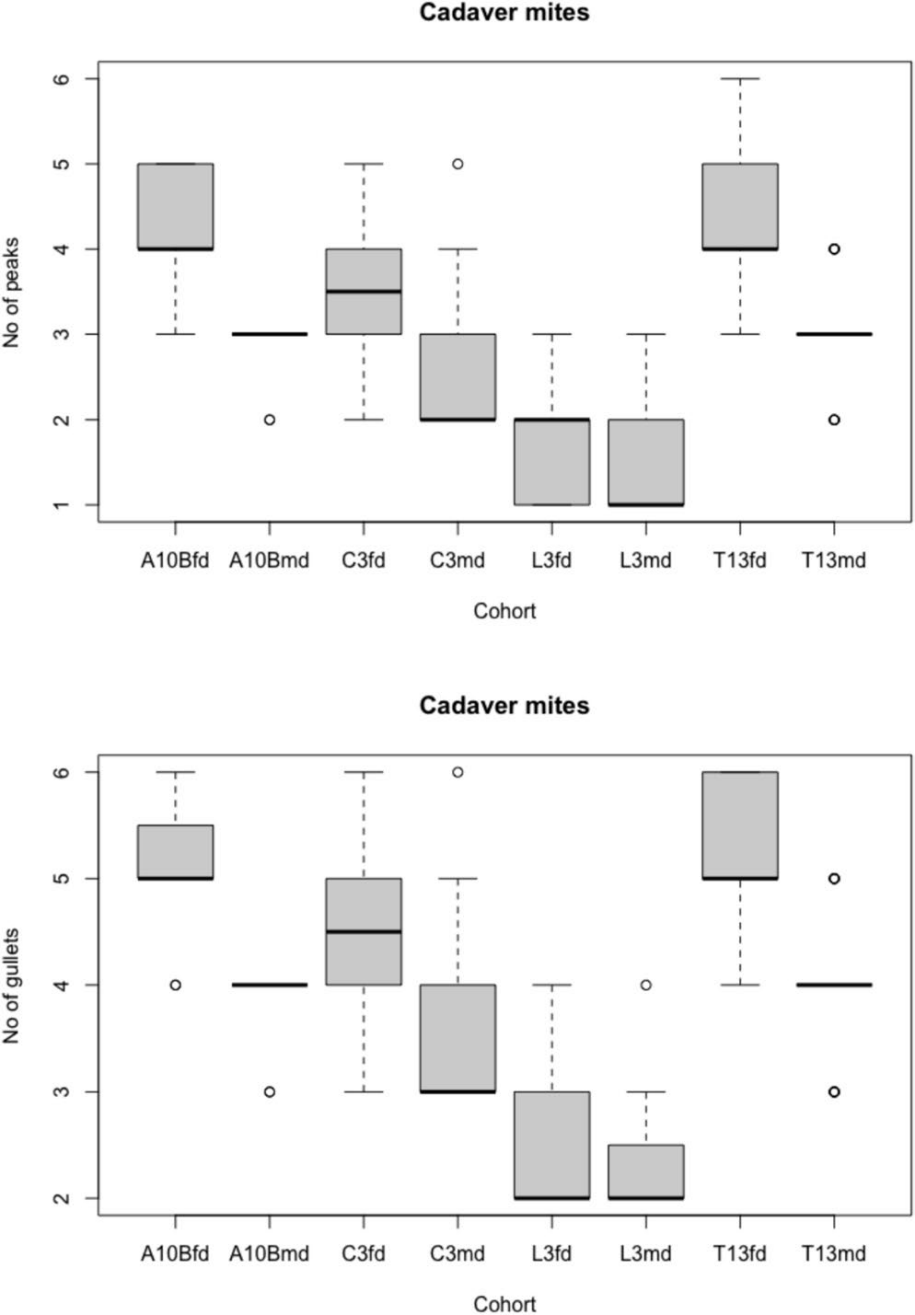
Box-whisker plot of number of peaks and gullets for each species and each digit (fd = fixed digit, md= moveable digit) for astigmatan individuals from species regularly found in cadavers. A10B = *Acarus siro*. C3 = *Sancassania berlesei*. L3 = *Lardoglyphus zacheri*. T13 = *Tyrophagus putrescentiae*. Solid line is mean. Quartiles and outliers highlighted

In the four cadaver-inhabiting species, usually the number of peaks on the fixed digit exceeds the number of peaks on the moveable digit (and similarly for the number of gullets on the fixed digit exceeding the number of gullets on the moveable digit, Fig. 10). In other words, fixed digits are more ‘toothy’. Note how the design of the rhizoglyphine acarid sits between the acarine acarids and the lardoglyphid.

**Fig. 11:**
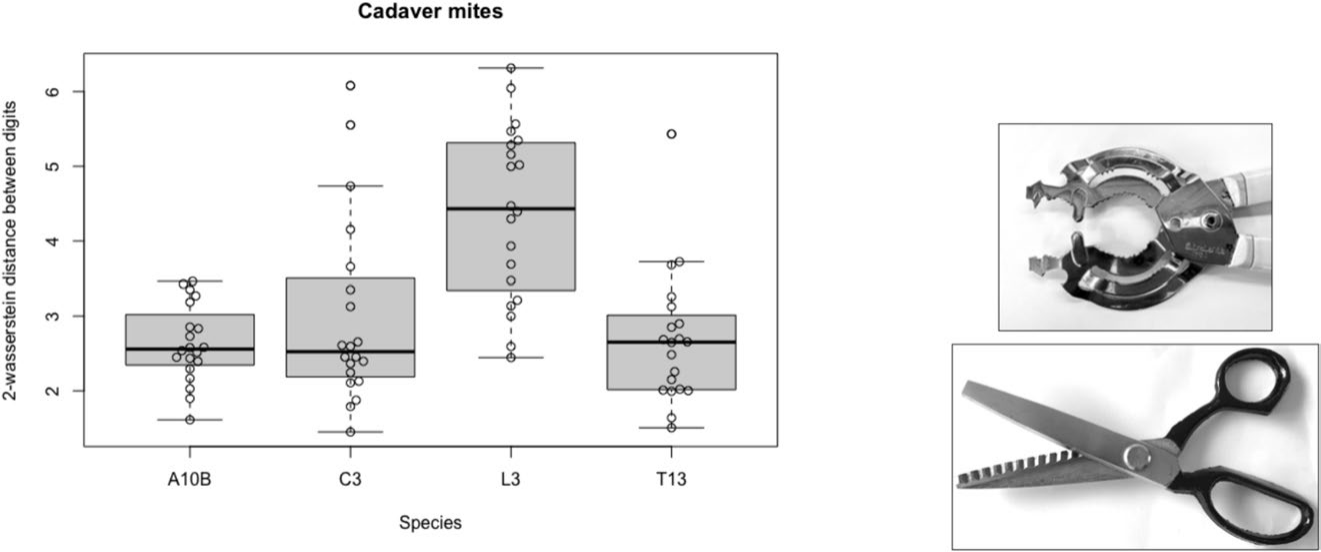
Left. Box-whisker and overlain Bee-swarm plot for 2-wasserstein distance between the set of asperities on chelal fixed and moveable digits for astigmatan individuals from species regularly found in cadavers. High values = poor occlusive fit between the digits. A10B = *Acarus siro*. L3 = *Lardoglyphus zacheri*. C3 = *Sancassania berlesei*. T13 = *Tyrophagus putrescentiae*. Open circles = individual specimen results. Solid line is mean. Quartiles highlighted. Right for comparison. Upper, domestic kitchen jar-opener tool with a 2-wasserstein distance $=4.53$ between its two sets of asperities. Lower, dress-making ‘pinking shears’ (from Bowman 2024a under Creative Commons licence) with exactly matching opposite asperities at distance $\approx 0$

In such mites one could consider that the digit tip is like an incisor $\equiv$ a chisel, then the distal slicing asperities $\equiv$ canines, before finally proximally then are found crunching asperities $\equiv$ premolar/molars. The exception are the lardoglyphids, which have a blade-like moveable digit surface potentially acting as a slicer (in Bowman 2025b). This disparity is confirmed by Fig. 11 where the lardoglyphid shows marked divergence between the occlusive design of its two digits. Note the greater variation amongst *Sancassania berlesei* C3.

**Fig. 12:**
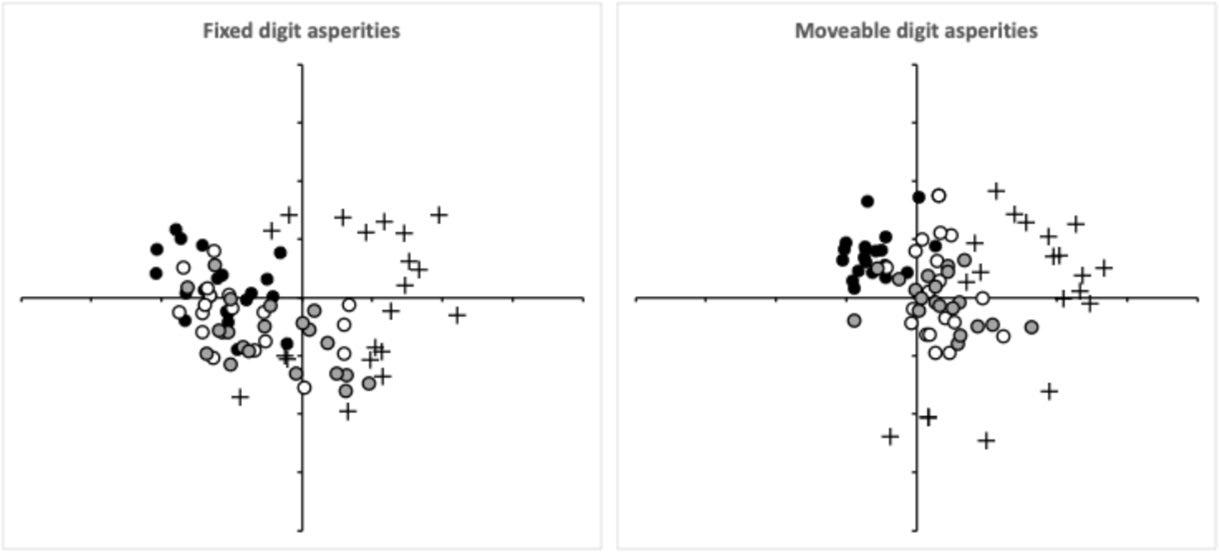
Two dimensional multidimensional scaling (with aspect ratio = 1, *stress* = 0.194) ordination of normalised 2-wasserstein distance between each digit to each digit across all individuals for species regularly found in cadavers. Left fixed digit asperities. Right moveable digit asperities. Black dots = *Acarus siro* A10B. Open circles = *Sancassania berlesei* C3. Crosses = *Lardoglyphus zacheri* L3. Grey dots = *Tyrophagus putrescentiae* T13

Comparing raw profile heights along the *L*2 digit reference axes can struggle because a small shift in the position of a theoretical valley, say, can dramatically change the outcome of a comparison. A ‘earth-mover’ cost avoids this bias (Morris et al. 2023). So, matching every fixed digit to every moveable digit across all species (and individuals) and calculating the normalised 2-wasserstein distance between them (i.e., comparing them in terms of their ‘bouquet’ of asperities), then performing an overall multidimensional scaling (MDS) ordination yields Fig. 12. Close inspection (or the plotting of the means, *not shown*) shows an upper left $\rightarrow$ lower right cline from *Acarus siro* A10B through the intermediate *Sancassania berlesei* C3 to *Tyrophagus putrescentiae* T13. This cline is well correlated with the change in surface roughness ($R_{q}$) of the profiles between the three species (*Acarus siro* A10B high roughness, *Tyrophagus putrescentiae* T13 low roughness, $r=0.862,0.909$ for fixed digit and moveable digit asperities respectively, *plots not shown*). Adjusting for any non-euclidearity would just accentuate this.

The unusualness of *Lardoglyphus zacheri* is driven by the difference to the other three species in their fixed digit peaks, their fixed digit gullets, their moveable digit peaks and their moveable digit gullets (*plots not shown*). Within a species there is a higher consistency of the design between the moveable and fixed digits for *Acarus siro* A10B and for *Lardoglyphus zacheri* L3 compared to the the lack of design ‘matching’ in *Sancassania berlesei* C3 and *Tyrophagus putrescentiae* T13 (*plots not shown*). So the relative design of the pair of digits in the former first two species (A10B & L3) is more coherent as a symmetrically patterned tool (like a nut/lobster-cracker or like umbilical cord scissors, Bowman 2024a, 2025b respectively) than the latter last two species (C3 & T13, the latter taxon more matching the design of steel cable-wire cutters Bowman 2025b).

The frequency of tooth type varies between species and between digits (Table 3). Fixed digits are again markedly ‘toothier’ (except for *Lardoglyphus zacheri* L3). Fixed digits invariably have fewer flat top (blade-like) teeth per digit on average. With the exception of *Lardoglyphus zacheri* L3, fixed digits have around twice the number of ATB-like teeth on average than their corresponding moveable digits. *Tyrophagus putrescentiae* T13 has an unusually ‘toothy’ moveable digit. The highest number on average for ATB-like teeth was for *Acarus siro* A10B, the lowest was for *Lardoglyphus zacheri* L3. The former would facilitate the dry crunching of material, the latter would be the pattern of a scissor blade for the slicing of pliable yielding material (soft flesh?). The highest number on average for flat top (blade-like) teeth was the distinctive fixed digit of *Tyrophagus putrescentiae* T13. Note that *Lardoglyphus zacheri* L3 has an unusually scissor blade-like moveable digit. ATB-like teeth in the lardoglyphid were exceedingly rare. The overall dental design of *Acarus siro* A10B and *Sancassania berlesei* C3 is broadly similar.

**Table 3:** Average number ($\mu$) of each tooth type (ATB versus FT) per chelal digit in astigmatans commonly occurring in cadavers. A10B = *Acarus siro*. C3 = *Sancassania berlesei* C3. L3 = *Lardoglyphus zacheri*. T13 = *Tyrophagus putrescentiae*. ATB = alternate top bevel-like. FT = flat top

Species	Digit	Tooth	$\mu$	Digit	Tooth	$\mu$
		type			type	
A10B						
	Moveable			Fixed		
		ATB	1.45		ATB	3.70
		FT	1.45		FT	0.65
C3						
	Moveable			Fixed		
		ATB	1.40		ATB	2.55
		FT	1.15		FT	0.90
L3						
	Moveable			Fixed		
		ATB	0.05		ATB	0.06
		FT	1.35		FT	1.05
T13						
	Moveable			Fixed		
		ATB	1.35		ATB	2.15
		FT	1.65		FT	2.05

The sequence of tooth type (anterior distal $\rightarrow$ posterior proximal) varies across the species and digit (Table 4) suggesting unique differentiation of chelae at the species level (even for *Acarus siro* A10B versus *Sancassania berlesei* C3). Optimal mastication thus must vary with the wetness of food material, with more concerted dry crushing by the acarine and more crush-slicing by the rhizoglyphine (e.g., consider masticating dry granola versus cooked moist oat porridge). Indeed, the chelal design of *Tyrophagus putrescentiae* T13 is rather like a ‘Swiss-Army pocket knife’ in its potential versatility (Bowman 2026).

**Table 4:** Probability of one tooth type (ATB versus FT) following another type (ATB versus FT) [anterior distally $\rightarrow$ posterior proximally] for chelal digits in astigmatans commonly occurring in cadavers. A10B = *Acarus siro*. C3 = *Sancassania berlesei* C3. L3 = *Lardoglyphus zacheri*. T13 = *Tyrophagus putrescentiae*. ATB = alternate top bevel-like. FT = flat top

Species	Digit	‘First’	Following		Digit	First	Following	
		type	types			type	types	
A10B								
	Moveable				Fixed			
			ATB	FT			ATB	FT
		ATB	0.227	0.773		ATB	0.912	0.088
		FT	0.313	0.688		FT	0.800	0.200
C3								
	Moveable				Fixed			
			ATB	FT			ATB	FT
		ATB	0.476	0.524		ATB	0.721	0.279
		FT	0.300	0.700		FT	0.333	0.677
L3								
	Moveable				Fixed			
			ATB	FT			ATB	FT
		ATB	0	0		ATB	0	1
		FT	0	1		FT	0.444	0.556
T13								
	Moveable				Fixed			
			ATB	FT			ATB	FT
		ATB	0.706	0.294		ATB	0.649	0.351
		FT	0.217	0.783		FT	0.444	0.556

**Fig. 13:**
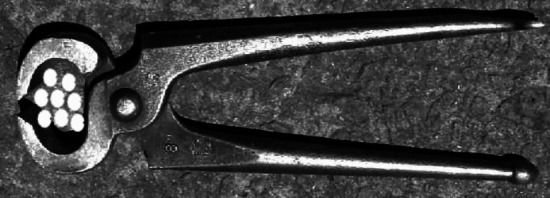
Each of the two ‘pockets’ in chela of *Lardoglyphus zacheri* L3 approximates the design of carpenter’s pincers used to grip behind objects (like nails) and then drag them out backwards (image amended under Creative Commons Licence from pincers.jpg by Securiger 23 Feb 2005 https://en.wikipedia.org/wiki/Pliers). White dots are myofibrils in cross-section, here 3 by 3 being the approximate fixed digit pocket size

What might *Lardoglyphus zacheri* enclose between its digits (i.e., prise-out and slice on chelal retraction)? Lardoglyphids are found infesting meat and fish products (Hughes 1976). Such products are rich in proteinaceous muscle tissues. Human muscle fibres typically range from $10-100\mu$m in diameter, with constituent myofibrils of $1-2\mu$m diameter (https://en.wikipedia.org/wiki/Myofibril). Now the average moveable digit mastication surface length ($x_{i_{e}}$) for *Lardoglyphus zacheri* L3 was $13.5\mu$m suggesting as a whole it could cut the smallest fibres or prise-out sets of myofibrils. In that way the digits of *Lardoglyphus zacheri* L3 partly approximate carpenters nail-drawing pliers (Fig. 13).

One should expect that other lardoglyphids share this design. They may also do slow-speed ‘skimming’ (see Bowman 2024a, b) given the small digit teeth proximally (Fig. 1). Indeed, each of the two fixed digit ‘pockets’ being around $4\mu$m wide would trap for slicing $2-4$ typical sized myofibrils.

### Commonalities with other species

**Fig. 14:**
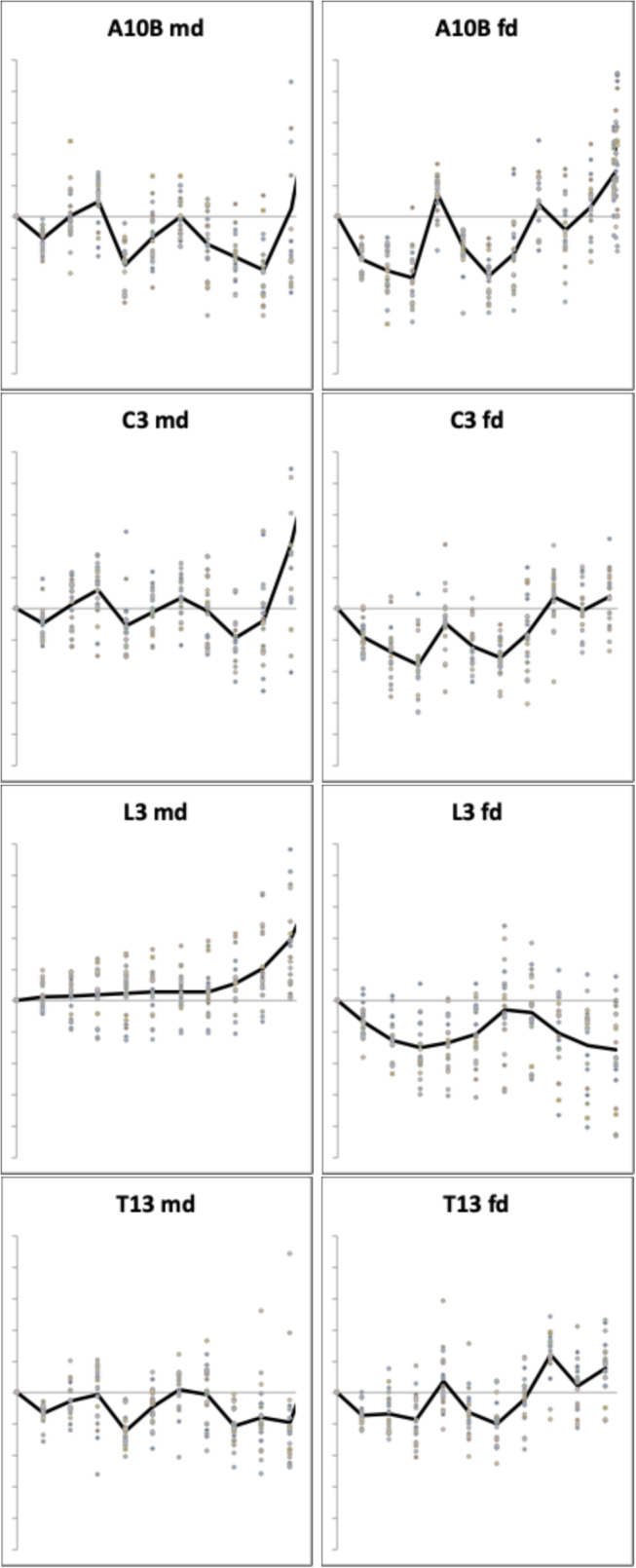
Mastication surface [*x*, *y*] ($2 \le k \le 11$) profiles in astigmatans commonly found in cadavers. $x_{k}$ locations fixed. A10B = *Acarus siro*. C3 = *Sancassania berlesei*. L3 = *Lardoglyphus zacheri*. T13 = *Tyrophagus putrescentiae*. md = moveable digit. fd = fixed digit. Grey dots individual specimens ($n=20$). Black line = average over individuals. *y*-axis exaggerated for pedagogy. Note: fixed digits are reflected vertically here i.e., a fixed digit gullet is where a corresponding moveable digit peak fits into on chelal occlusion. *Lardoglyphus zacheri* L3 is very different to the other species

The astigmatan mastication surfaces can be compared using the $\Omega$ methods from Bowman (2024b, 2025a). The mastication surface profiles for the cadaver mites are shown in Fig. 14.

This nicely illustrates the declining roughness of digits *Acarus siro* A10B $\rightarrow$ *Sancassania berlesei* C3 $\rightarrow$ *Tyrophagus putrescentiae* T13 and their occlusal opposability. It also confirms the scimitar-like moveable digit blade of the lardoglyphid and its two ‘pocketed’ fixed digit design. Note the complicated proximal ‘notch’ first described in Bowman (2024b) for *Tyrophagus putrescentiae* T13.

**Fig. 15:**
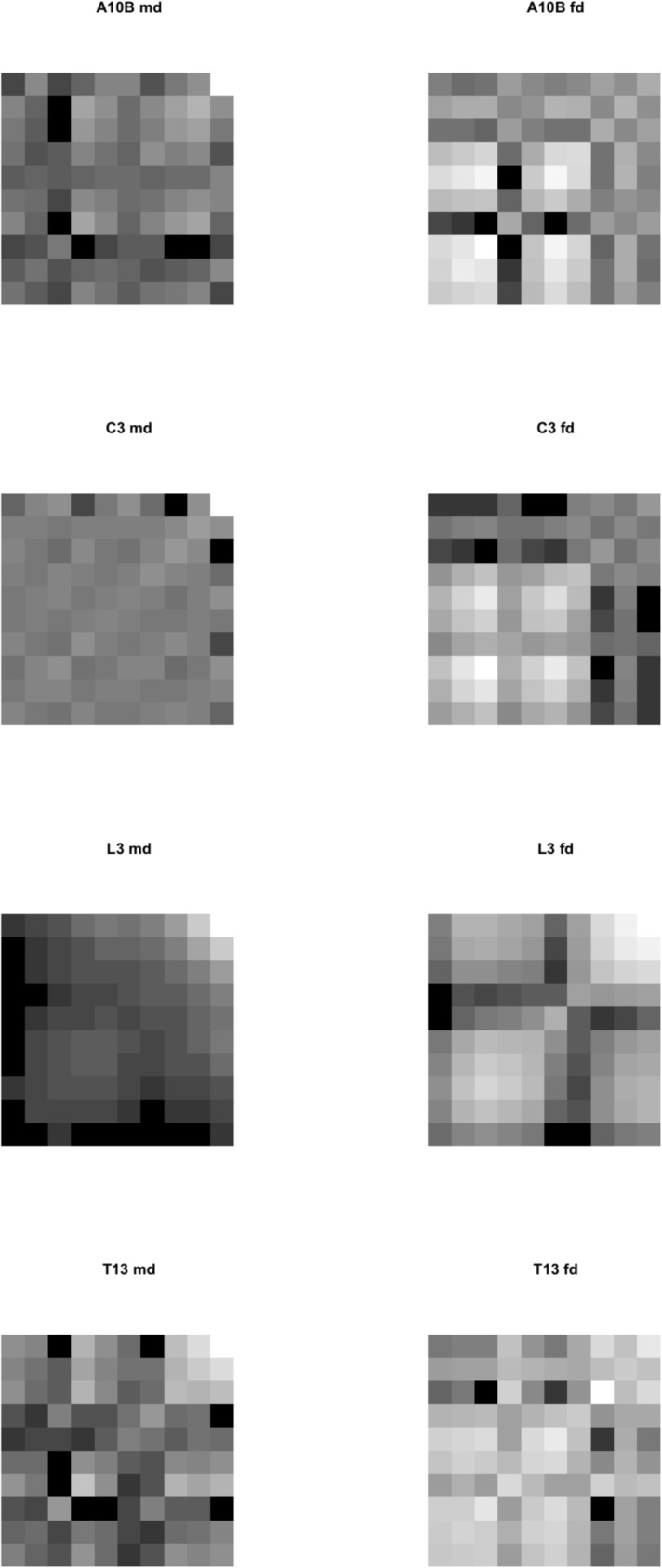
$\Omega$ matrices (Bowman 2024b, 2025a) for the mastication surface [*x*, *y*] profile ($2 \le i \le 11$) in astigmatans commonly found in cadavers. Darkness represent magnitude (black = min $\equiv$ low co-occurrence, white = max $\equiv$ high co-occurrence). A10B = *Acarus siro*. C3 = *Sancassania berlesei*. L3 = *Lardoglyphus zacheri*, note two clear ‘pockets’ indicated along diagonal of fixed digit. T13 = *Tyrophagus putrescentiae*. md = moveable digit. fd = fixed digit. Origin = digit tip at bottom left of each sub-panel. End of mastication surface ($x_{i_{e}}$) top right

The corresponding $\Omega$ matrices, shaded by matrix values for each are shown in Fig. 15. $\Omega$ matrices are positive semi-definite being an average quadratic function of digit profile height measurements projected onto their reference axis (Bowman 2024b). Note that the moveable digit pattern varies across all four species. In contrast, note also the distinct fixed digit patterns of co-occurrence over a common general theme for *Acarus siro* A10B , *Sancassania berlesei* C3 and *Tyrophagus putrescentiae* T13. On the contrary, the *Lardoglyphus zacheri* L3 fixed digit is markedly different.

**Fig. 16:**
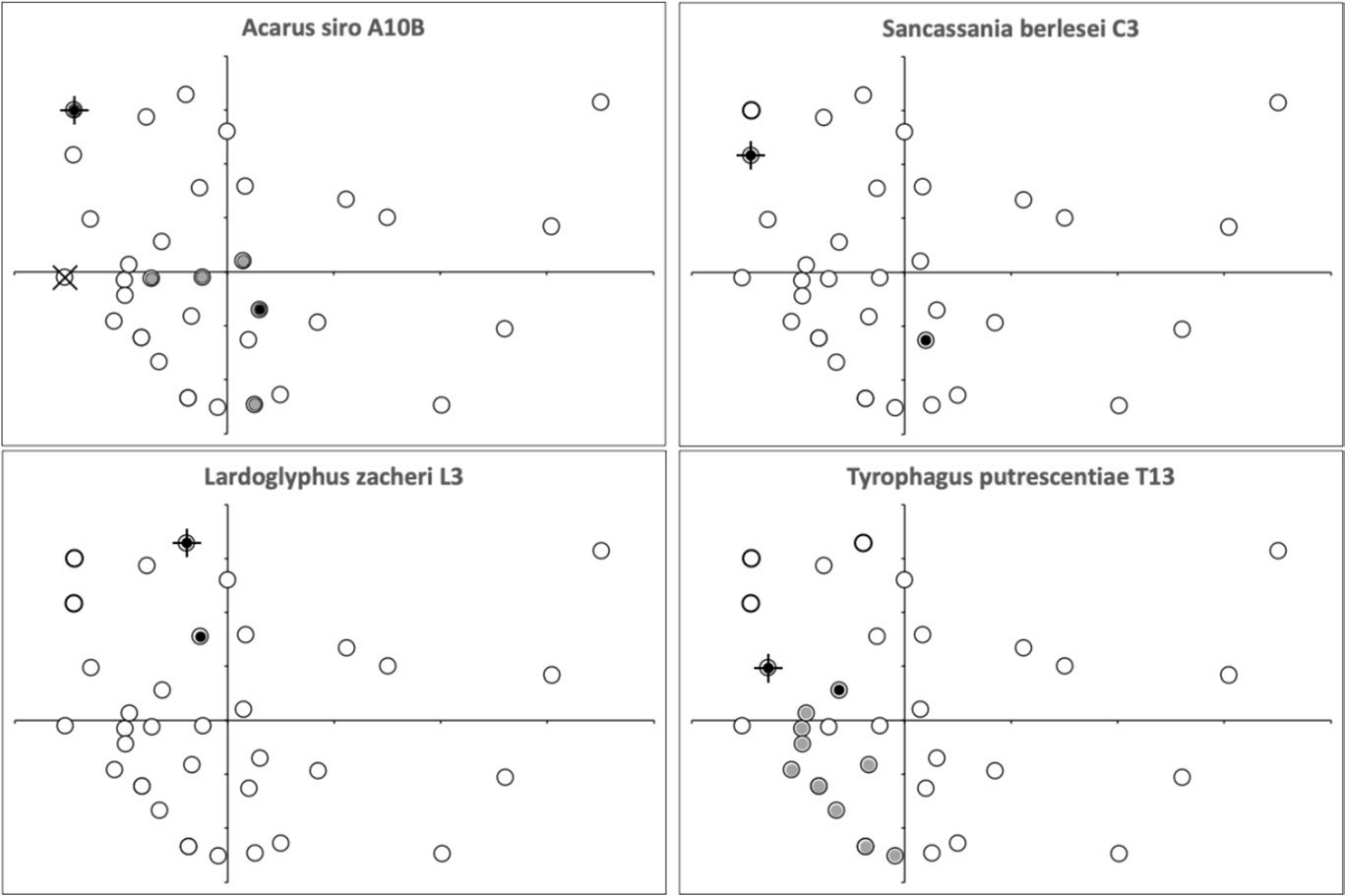
Multidimensional scaling ordination of normalised Wasserstein metric between digit profiles (post-digit tip to overall end of mastication surface, $stress=0.151$). Black dots = specified cadaver astigmatan species. Grey dots = moveable digit profiles of other out-group species of astigmatans in that genus from Bowman (2025a, 2026). Cross in top left sub-figure = *Carpoglyphus lactis* CA4 from Bowman (2024b). Plus = fixed digit for that cadaver astigmatan species

Calculating the normalised 2-wasserstein distance for post-tip profile data locations $2 \le i \le 11$ for the fixed and moveable digits of the cadaver mites and the moveable digits of all bird nest inhabiting species from Bowman (2025a, 2026) and out group comparators, followed by an MDS ordination yields Fig. 16 where key digits are highlighted.

**Fig. 17:**
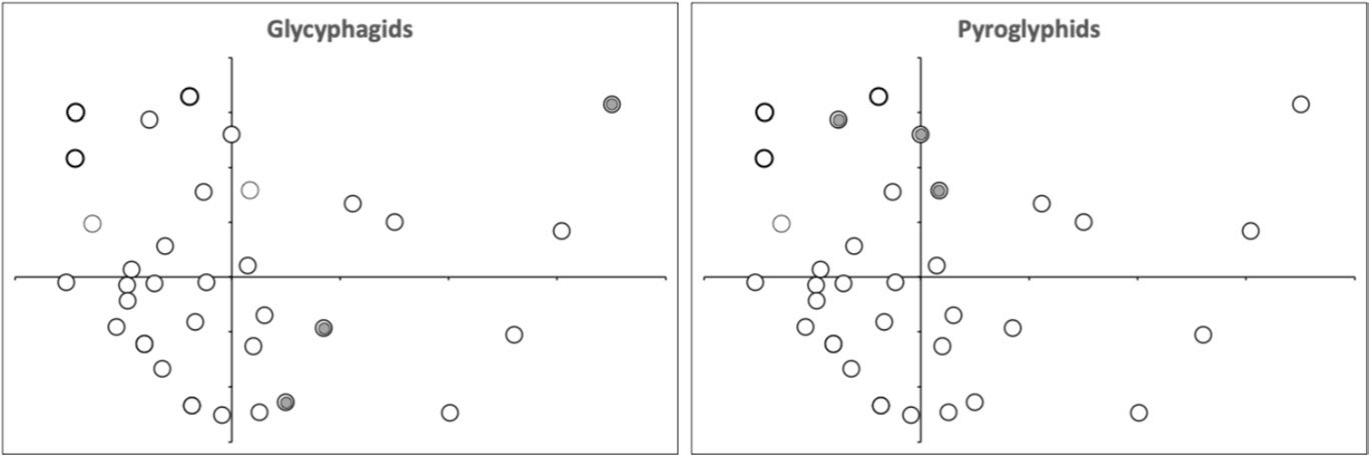
Multidimensional scaling ordination of normalised Wasserstein metric between digit profiles (post-digit tip to overall end of mastication surface). See Fig. 16. Grey dots = moveable digit profiles of out-group species of astigmatans in that family from Bowman (2025a, 2026). Extreme top right glycyphagid = *Glycycometrus hughesae* G3. Pyroglyphid in top left quadrant of each subfigure is *Dermatophagoides microceras* D5

This points to distinct clusters of mastication surface design for *Acarus* spp. and *Tyrophagus* spp. within the overall digit designs found in Bowman (2021a). Moreover, glycyphagids and pyroglyphids are also again distinct (Fig. 17).

**Fig. 18:**
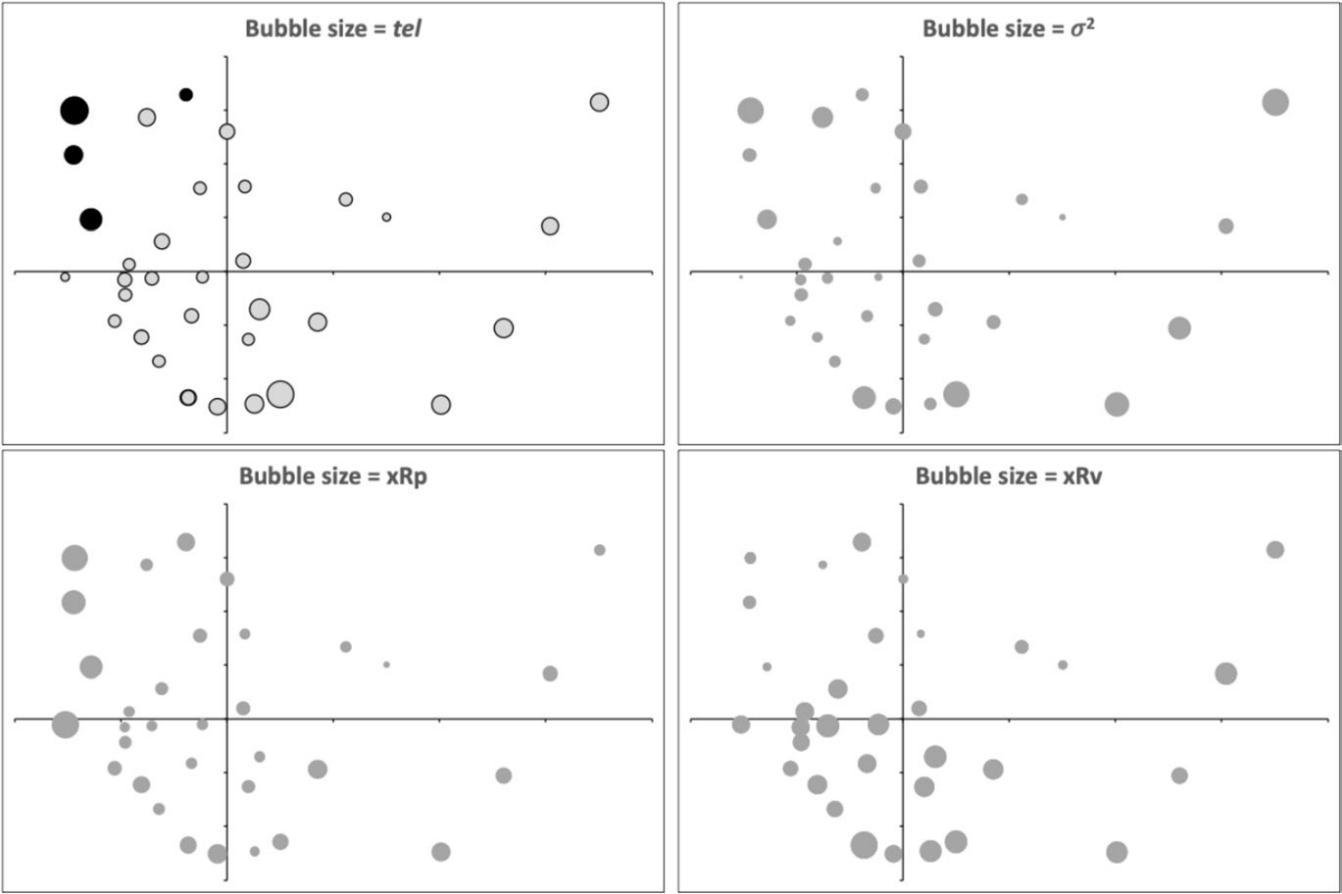
Bubble plots of multidimensional scaling ordination of normalised Wasserstein metric between digit profiles (post-digit tip to overall end of mastication surface). See Fig. 16. Grey dots = moveable digit profiles of out-group species of astigmatans from Bowman (2025a, 2026). Black dots = fixed digit (only highlighted in the top left sub-panel)

The location of each taxon (and digit type) is related to the *tel* point-process stretch measure (Bowman 2024a) for that digit profile (Fig 18). This can be traced to a higher variance in profile heights ($\sigma ^{2}$, Bowman 2024a).

Dissecting this into the location of maximum peak (*xRp*) and the location of maximum gullet (or valley, *xRv*) from Bowman (2024a), shows that the location on the ordination reflects the ‘toothiness’ of a digit and the pattern of that ’toothiness" (Bowman 2024a). This is instrumental in the Type of digit (A = ‘tearing hook-like’ versus B = ‘nibbling’ digit morphotypes) and their claw-length equivalent (*CLE*) values. Note that the fixed digits cluster to the top left, distinct as expected, as if their asperities match on occlusion with their respective moveable digits, they should be distinct (indeed like ’opposites attract’, profile deflections should be inverted). They also sit with the unusual moveable digit of *Dermatophagoides microceras* D5.

## Conclusion

The chelae of cadaver-inhabiting astigmatans are rationally designed to attack material likely to be present at each stage of body decomposition and so to help these cadaver astigmatans to avoid trophic competition. All in all, fixed digit design variation in free-living astigmatans is all about ‘peakiness’ for biting and moveable digit designs are all about differing ’gullet-ness’ for material ‘scooping’ (Fig 18).

There are plausible rationales for why different astigmatans are found in different types of carcasses at different times. The above results indicate that the observed sequence in which at least two of the four key astigmatan mites appear on unenclosed decomposing carcasses is reflected in their different trophic design for the availability of likely food at that point.

- As an agricultural pest *Acarus siro* pioneers grain infestations preferentially attacking fresh high protein sources (like wheatgerm). A dead body provides such a source for mites of a necrophagous habit throughout the whole time course of decay once it is open to attack. Native proteins (like in dried grain) can be quite hard to chew - requiring this ’demolition-excavator’ design (Bowman 2021a) to break-in and effectively triturate them.
- *Tyrophagus putrescentiae* is a cosmopolitan species found in a range of habitats, including grasslands, old hay, mushrooms, and the nest of bees and ducks. As an infestor of stored products, it feeds on the fungi that grow on high protein, high fat content foodstuffs. The results in Bowman (2023) show it should be able to grasp yeasts, spores and mycelial hyphae which is consilient with *Tyrophagus putrescentiae* being regarded as a saprophagous burrowing ’browser/gleaner’ generalist. That is, a fragmentary feeder with a small bite size, grabbing relatively small and relatively soft food morsels that are selectively squashed using its moderate chelal output *F*2 force. A late stage decayed body should have residual tissues permeated by much soil microflora consilient with this. Future detailed work should confirm exactly when fungal colonisation of the body surface occurs and why interstitial fragmentary feeding mites can invade and prosper as the body switches from bloated stage to the putrefying conditions. Is this the time of major fungal invasion inside the carcass? Do fungal feeding mesostigmatids invade at this time too?

At what point might any of the four species be preferentially utilising fat in the carcass?

*Sancassania* spp. consume mummified cadavers which will contain saponified tissues to a point that they are very well preserved. Saponified tissues are just fat transformed into soap so somehow the former species must have a somehow similar bite as *Lardoglyphus* spp. Perhaps *Sancassania* spp. prefer ‘harder’ soaps (derived from saturated body fats) while the latter species prefer ’softer’ more unsaturated soaps (perhaps derived from brains and nerve tissues)? For sure, even lardoglyphids can be reared upon a variety of food material (Rady et al. 2018). However, archetypically *Lardoglyphus zacheri* is a soft small food morsel design specialism of the *Tyrophagus longior* surface omnivore variant Bowman (2021a). It is designed for taking tiny mouthfuls from little chunks of food requiring little effort to crush. As a ’soft slicer’, is this why it first appears at the bloated stage when tissues may be inflating?

Nevertheless, while the cadaver inhabiting species are distinct from each other, each does share trophic features with their non-cadaver inhabiting congeners (Bowman 2021a, 2026). This is to be expected as the genus is the taxonomic rank between family and species and taxonomists group organisms into a genus if they share many structural similarities and are very closely related. Members of a genus (or sub-family) being more closely related to each other than they are to other genera in the same family. So why particular species (within a genus) are successful in cadavers rather than their close out-group relatives must be down to other physiological or behavioural adaptations.

## Future work

This study answers many of the specific questions posed about astigmatan mastication surfaces by Bowman (2021a). Formal statistical hypothesis testing using other routines in the Riemannian package for R awaits larger samples over more species. Other distance measures like Jeffrey’s or Kullback-Leibler divergences could be used in follow up work. However, for sure an accepted classification of digit gullet shapes needs to be established for taxonomists to score their specimens.

Some astigmatans can be found internally in living human beings (Li and Wang 2000,Li et al. 2003a, b), some getting there via ingestion and some via invasion. If alive, do these internal mites feed in a similar way to the cadaver mites? Do forensic acarologists find particular species of mites in certain decaying organs of corpses to match?

Although various papers explain in which stages of body decomposition astigmatans thrive (i.e., that they are eating their way through), scanning electron microscopy of cadaver skin and tissue surfaces attacked by mites would help validate how their digits affect the material. How much damage is caused by *Lardoglyphus zacheri* could be compared to other high protein feeding lardoglyphids like *Lardoglyphus konoi*, (for their taxonomic distinction see Hughes 1956). More recently further fish tissue infesting species (Olsen 1982) and human mummy inhabiting (Baker 1990) have been described.

Moreover, more work upon the time course of chemical and structural changes to protein and fat in carcasses would be useful. Micro-penetrometer analyses would be useful, to validate how easy burrowing may or may not be for free-living astigmatans. Lipase assays (over time) would be useful for each mite species to see if this could be related to fat degradation in the carcass. Confirmatory astigmatan mite gut analyses and further protein and fat degrading enzyme assays could then be useful (especially in *Sancassania berlesei*) in critically proving what the differently designed mites are exactly eating at what time of the carcass decomposition.

There is much left for forensic acarologists to do!

## Data Availability

Alcohol preserved samples of mite laboratory cultures have been deposited at: the British Museum (Natural History), London UK under accession number AQ ZOO 2020-78, and the Museum of Zoology, University of Michigan, Ann Arbor, MI USA under accession number ITR-UMMZ-I-2020-018. All new data collected, generated and analysed at the Oxford Centre for Industrial and Applied Mathematics (by CEB), plus any model specifications are included in this or previously published articles or in compliance with EPSRC’s open access initiative are available from https://dx.doi.org/10.5287/ora-kzgngvyjj

## References

[CR1] Akimov IA (1985) Biological foundations of harmfulness in acaroid mites. Naukova Dumka, Kiev, p 160. in Russian

[CR2] Akimov IA, Gaichenko VA (1976) The principle of action of the claws of the chelicerae in mites of the families Acaridae Leach, 1816 and Glyciphagidae Berlese, 1923 in connection with their adaptation to different food substrates. Dopovididi Akademiyi Nauk Ukrayins‘koy RSR, seriya B. Heolohichni, khimichni ta biolohichni nauky 0(4):352-355

[CR3] Baker AS (1990) Two new species of Lardoglyphus Oudemans (Acari: Lardoglyphidae) found in the gut contents of human mummies. J Stored Prod Res 26(3):139–147. 10.1016/0022-474X(90)90014-J

[CR4] Baker AS (2009) Acari in archaeology. Exp Appl Acarol 49:147–160. 10.1007/s10493-009-9271-1 19513808 10.1007/s10493-009-9271-1

[CR5] Bhatia R, Jain T, Lim Y (2017) On the Bures-Wasserstein distance between positive definite matrices. arXiv:1712.01504v1

[CR6] Bhatia R, Jain T, Lim Y (2019) On the Bures-Wasserstein distance between positive definite matrices. Expo Math 37:165–191. 10.1016/j.exmath.2018.01.002

[CR7] Bookstein FL (1986) Size and shapes spaces for landmark data in two dimensions (with discussion). Stat Sci 1:181–242

[CR8] Bowman CE (1981) Hide protease in stored product mites (Astigmata: Acaridae) Comparative Biochemistry and Physiology. B Comparative Biochem 70(4):803–805

[CR9] Bowman CE (2020) Feeding design in free-living mesostigmatid chelicerae (Acari: Anactinotrichida). Exp Appl Acarol 84(1):1–119. 10.1007/s10493-021-00612-8 10.1007/s10493-021-00612-8 PMC8085810 33929649

[CR10] Bowman CE (2021a) Cheliceral chelal design in Astigmatid mites. Exp Appl Acarol 84(2):271–363. 10.1007/s10493-021-00625-3 33988815 10.1007/s10493-021-00625-3 PMC8189993

[CR11] Bowman CE (2021b) Could the acarid mite Tyrophagus putrescentiae (Schrank) be used as an environmental thermometer? Int J Acarology 47(2):107–118. 10.1080/01647954.2021.1877350

[CR12] Bowman CE (2023) Variation in the trophic morphology of Astigmatid mites common in UK beehives. Acarologia 63(Suppl):4–16. 10.24349/z9n6-u3t3

[CR13] Bowman CE (2024a) Do astigmatid teeth matter: a tribological review of cheliceral chelae in co-occuring mites from UK beehives. Exp Appl Acarol 92(4):567–686. 10.1007/s10493-023-00876-2 38639851 10.1007/s10493-023-00876-2 PMC11636773

[CR14] Bowman CE (2024b) Transitional chelal digit patterns in saprophagous astigmatan mites. Exp Appl Acarol 92(4):687–737. 10.1007/s10493-024-00907-6 38622432 10.1007/s10493-024-00907-6 PMC11065788

[CR15] Bowman CE (2025a) Transecting and contrasting the feeding designs of the astigmatan community from bird nests. Exp Appl Acarol 94:52. 10.1007/s10493-025-01014-w 40232569 10.1007/s10493-025-01014-w PMC12000161

[CR16] Bowman CE (2025b) Further mechanistic insights into the trophic design of free-living mites. (presented at 10th European Association of Acarologists (EurAAc) Symposium - 2–6 September 2024, Athens, Greece) Acarologia 65(3), 677–707. 10.24349/sah5-bh2d

[CR17] Bowman CE (2026) Comparative dentition in free-living bird nest astigmatan mites. Exp Appl Acarol 96(9):1–91. 10.1007/s10493-025-01091-x 10.1007/s10493-025-01091-x PMC12738647 41441919

[CR18] Braig HR, Perotti MA (2009) Carcases and mites. Exper Appl Acarol 49(1–2):45–84. 10.1007/s10493-009-9287-6 19629724 10.1007/s10493-009-9287-6

[CR19] Chmielewski W (2003) Effect of buckwheat sprout intake on population increase of Caloglyphus berlesei (Michael) (Acari: Acaridae). Fagopyrum 20:85–88

[CR20] Cunnington AM (1966) Physical limits for complete development of the grain mite, Acarus siro L. (Acarina, Acaridae), in relation to its world distribution. J Appl Ecol 2(2):295–306. 10.2307/2401481

[CR21] Deeming DC, Gilchrist R, Szafraniec M, Pollins JM (2020) Water vapour conductance of passerine nest walls. Acta Ornithologica 55:13–21. 10.3161/00016454AO2020.55.1.002

[CR22] Doaa AE-E-A, Noureldin G, Mohamed O (2014) Effects of temperature on the life-history traits of Sancassania (Caloglyphus) berlesei (Acari: Astigmatina: Acaridae) feeding on root-knot nematodes, Meloidogyne spp. (Nematoda: Meloidogynidae). Exper Appl Acarol 64(3):299–307. 10.1007/s10493-014-9826-7 24923664 10.1007/s10493-014-9826-7

[CR23] Erban T, Rybanska D, Hubert J (2015) Population Growth of the Generalist Mite Tyrophagus putrescentiae (Acari: Acaridida) Following Adaptation to High- or Low-Fat and High- or Low-Protein Diets and the Effect of Dietary Switch. Physiol Ecol 44(6):1599–1604 10.1093/ee/nvv129 26314031

[CR24] García-del Río M, Cantarero A, Castaño-Vázquez F, Merino Y, García-Velasco J, Merino S (2024) Experimental manipulation of nest temperature and relative humidity reduces ectoparasites and affects body condition of Blue Tits (Cyanistes caeruleus). Ibis 167(1):212–224. 10.1111/ibi.13346

[CR25] Goff ML (2009) Early post-mortem changes and stages of decomposition in exposed cadavers. Exp Appl Acarol 49(1–2):21–36. 10.1007/s10493-009-9284-9 19554461 10.1007/s10493-009-9284-9

[CR26] Griffiths DA (1979) The morpho-species and its relationship to the biological species in the genus Tyrophagus (Acaridae: Acarina). In: Rodriguez JG (ed) Recent advances in acarology, vol 1. Academic Press, New York, pp 199–212

[CR27] Hubert J, Pekár S, Aulický R, Nesvorná M, Stejskal V (2013) The effect of stored barley cultivars, temperature and humidity on population increase of Acarus siro, Lepidoglyphus destructor and Tyrophagus putrescentiae. Exper Appl Acarol 60:241–252. 10.1007/s10493-012-9639-5 23192331 10.1007/s10493-012-9639-5

[CR28] Hughes AM (1956) The mite genus Lardoglyphus Oudemans 1927 (= Hoshikadana Sasa and Asanuma 1951). Zoologische Mededelingen 34(20):271–285

[CR29] Hughes AM (1976) The mites of stored food and houses. HMSO, 2nd ed. Ministry of Agriculture, Fisheries and Food Technical Bulletin vol 9, pp 1-400

[CR30] Iverson K, Oconnor BM, Ochoa R, Heckmann R (1996) Lardoglyphus zacheri (Acari: Lardoglyphidae), a Pest of Museum Dermestid Colonies, with Observations on Its Natural Ecology and Distribution. Ann Entomol Soc Am 89(4):544–549. 10.1093/aesa/89.4.544

[CR31] Jayas DS, White NDG, Muir WE (1995) Stored-Grain Ecosystems. (Papers presented at an International Symposium on Stored Grain Ecosystems, held in Winnipeg, Manitoba, Canada, from June 7-10, 1992) Marcel Dekker p 757

[CR32] Johnston DE (1965) Comparative studies on the mouth-parts of the mites of the suborder Acaridei (Acari). Unpublished PhD dissertation, Ohio State University

[CR33] Jónsdóttir GO, Ingimarsson F, Snorrason SS, Steele SE, Pálsson A (2026) Variation of Tooth Traits in Ecologically Specialized and Sympatric Morphs. Evol Biol 53:67–84. 10.1007/s11692-026-09665-2

[CR34] Karagoz M, Gulcu B, Cakmak I, Kaya HK, Hazir S, Acari (2007) Acaridae) Exp Appl Acarol 43(2):85–85. 10.1007/s10493-007-9105-y 17924198 10.1007/s10493-007-9105-y

[CR35] Kaur H, Bala M, Kaur N (2019) First record of Astigmatid mites (Acari: Sarcoptiformes) from animal carcasses of Punjab(India). J Emerging Technol Innov Res 6(6):65–73

[CR36] Krishnan K, Campbell YL, To KV, Lima G, Byron MD, Zhang X, Hendrix JD, Shao W, Cord CL, Crist CA, Phillips TW, Schilling MW (2019) Effects of temperature, relative humidity, and protective netting on Tyrophagus putrescentiae (schrank) (sarcoptiformes: Acaridae) infestation, fungal growth, and product quality of cave-aged Cheddar cheese. J Stored Prod Res 83:44–53. 10.1016/j.jspr.2019.05.014

[CR37] Leclercq M (1974) Entomologie et médecine légale Etude des insectes et acariens nécrophages pour déterminer la date de la mort. Spectrum int 17(6):1–7

[CR38] Leclercq M, Verstraeten C (1988) Entomologie et Médecine légale. Datation de la mort. Acariens trouvés sur des cadavres humains. Bulletin et annales de la Société royale belge d’entomologie 124:195–200

[CR39] Li C-P, Wang J (2000) Intestinal acariasis in Anhui Province. World J Gasterenterol 6(4):597–600. 10.3748/wjg.v6.i4.597 10.3748/wjg.v6.i4.597 PMC4723566 11819656

[CR40] Li C-P, Cui Y-B, Wang J, Yang Q-G, Tian Y (2003a) Acaroid mite, intestinal and urinary acariasis. World J Gastroenterol 9(4):874–877. 10.3748/wjg.v9.i4.874 12679953 10.3748/wjg.v9.i4.874 PMC4611470

[CR41] Li C-P, Cui Y-B, Wang J, Yang Q-G, Tian Y (2003b) Diarrhea and acaroid mites: A clinical study. World J Gastroenterol 9(7):1621–1624. 10.3748/wjg.v9.i7.1621 12854179 10.3748/wjg.v9.i7.1621 PMC4615520

[CR42] Mègnin JP (1894) La faune des cadavres-application de l‘entomologie a la médecine légale. Gauthier-Villars et fils

[CR43] Mohan S (2023) A note on the Bures-Wasserstein metric. arXiv:2303.03883v1

[CR44] Morris MJ, Lipp AG, Roberts GG (2023) Towards inverse modeling of landscapes using the Wasserstein distance. Geophys Phys Lett 50(14):e2023GL103880. 10.1029/2023GL103880

[CR45] Nader R, Bretto A, Mourad B, Abbas H (2019) On the positive semi-definite property of similarity matrices. Theoret Comput Sci 755:13–28. 10.1016/j.tcs.2018.06.052

[CR46] OConnor BM (2009) Astigmatid mites (Acari: Sarcoptiformes) of forensic interest. Exp Appl Acarol 49(1–2):125–33. 10.1007/s10493-009-9270-2 19609687 10.1007/s10493-009-9270-2

[CR47] Olsen AR (1982)A new pest of dried fish from the Orient, Lardoglyphus angelinae new sp. (Acarina:Acaridae). J Stored Products Res 18(4):181–188. 10.1016/0022-474X(82)90029-7

[CR48] Perotti MA (2009) Mègnin re-analysed: the case of the newborn baby girl, Paris, 1878. Exp Appl Acarol 49(1–2):37–44. 10.1007/s10493-009-9279-6 19557528 10.1007/s10493-009-9279-6

[CR49] Perotti MA, Braig HK (2022) The arthropods of corpses from above ground and from deep below. Zoosymposia 22:149–152. 10.11646/zoosymposia.22.1.97

[CR50] Perotti MA, Braig HR, Goff ML (2010) Phoretic Mites and Carcasses: Acari Transported by Organisms Associated with Animal and Human Decomposition. In: Amendt J (eds) Current Concepts in Forensic Entomology, pp 69-91, Springer Science + Business Media B.V. 10.1007/978-1-4020-9684-6_5

[CR51] Rady GH, Metwally AM, Mohamed GR, Ahmed N, Nagah AM (2018) Biological studies on only one mite species Lardoglyphus sp. belong to astigmatid mites under the laboratory conditions. Middle East J Agri Res 7(4):1710–1716

[CR52] Rai JK, Amendt J, Bernhardt V, Pasquerault T, Lindström A, Perotti AM (2020) Mites (Acari) as a relevant tool in trace evidence and postmortem analyses of buried corpses. J Forensic Sci 65(6):2174–2183. 10.1111/1556-4029.14506 32717143 10.1111/1556-4029.14506

[CR53] Rai JS, Pickles BJ, Perotti MA (2021) Assemblages of Acari in shallow burials: mites as markers of the burial environment, of the stage of decay and of body-cadaver regions. Exper Appl Acarol 85:247–276. 10.1007/s10493-021-00663-x 34622362 10.1007/s10493-021-00663-x PMC8604864

[CR54] Rivard I (1961) Influence of temperature and humidity on longevity, fecundity, and rate of increase of the mite Tyrophagus putrescentiae (Schrank) (Acarina: Acaridae) reared on mold cultures. Can J Zool 39(4):419–426. 10.1139/z61-081

[CR55] Rodriguez JG, Stepien ZA (1973) Biology and Population Dynamics of Caloglyphus berlesei (Michael) (Acarina: Acaridae) in Xenic Diet. J Kans Entomol Soc 46(2):176–183. http://www.jstor.org/stable/25082564

[CR56] Saloña MI, Moraza ML, Carles-Tolrá M, Iraola V, Bahillo P, Yélamos T, Outerelo R, Alcaraz R (2010) Searching the soil: forensic importance of edaphic fauna after the removal of a corpse. J Forensic Sci 55(6):1652–1655. 10.1111/j.1556-4029.2010.01506.x 20666921 10.1111/j.1556-4029.2010.01506.x

[CR57] Saloña Bordas MI, Perotti MA (2020) First record of Lardoglyphus zacheri (Acari, Lardoglyphidae) in the Iberian Peninsula and new observations on its insect carriers. Syst Appl Acarol 25(3):412–419. 10.11158/saa.25.3.3

[CR58] Sinha RN (1964) Ecological relationships of stored-products mites and seed borne fungi. Acarologia 6:372–389

[CR59] Solarz K, Paja̧k C, Pawełczyk O, Bobiński R, Ciechacka M, Dutka M, Ulman-Włodarz I (2022) Mites occurring in farm buildings as allergic agents and indicators in forensic analyses. Acarologia 62(1):36–47. 10.24349/gsxm-p9jj

[CR60] Szelecz I, Lösch S, Seppey CVW, Lara E, Singer D, Sorge F, Tschui J, Perotti MA, Mitchell EAD (2018) Comparative analysis of bones, mites, soil chemistry, nematodes and soil micro-eukaryotes from a suspected homicide to estimate the post-mortem interval. Sci Rep 8:25. 10.1038/s41598-017-18179-z 29311698 10.1038/s41598-017-18179-z PMC5758714

